# Nontargeted Analysis Reveals Organic Compounds That Drive Oxidative Potential in Ambient Particulate Matter

**DOI:** 10.1021/acs.est.5c07847

**Published:** 2026-02-23

**Authors:** Anna Breuninger, Alexander Schmidt, Florian Ungeheuer, Lingli Zhou, Jialiang Ma, Sarah S. Steimer, Alexander L. Vogel

**Affiliations:** † Institute for Atmospheric and Environmental Sciences, 9173 Goethe University Frankfurt, 60438 Frankfurt am Main, Germany; ‡ Frankfurt Isotope and Element Research Centre (FIERCE), 124117 Goethe University Frankfurt, 60438 Frankfurt am Main, Germany; § Department of Environmental Sciences, 7675 Stockholm University, 11419 Stockholm, Sweden; ∥ Institute for Geosciences, 9173 Goethe University Frankfurt, 60438 Frankfurt am Main, Germany; ⊥ 251404 South China Institute of Environmental Sciences, Ministry of Ecology and Environment, Guangzhou 510655, China

**Keywords:** particulate matter, organic aerosol, oxidative potential, nontargeted analysis, urban aerosol, molecular composition, mass spectrometry, hierarchical cluster analysis

## Abstract

Air pollution adversely affects human health, with studies consistently linking it to exposure to particulate matter. However, differential toxicity remains to be established to improve monitoring and mitigation efforts. This study examines the oxidative potential (OP) of fine particulate matter using the dithiothreitol (DTT) assay as a proxy for toxicity. We analyzed 42 ambient particle filter samples from Frankfurt, Germany, and Beijing, China, focusing on water-soluble organic compounds. The aqueous extracts were analyzed for metal content via inductively coupled plasma–mass spectrometry and for organic compounds through ultrahigh performance liquid chromatography–high-resolution mass spectrometry. Utilizing a chelating agent to remove metals allowed separation of the effects of bulk aerosol and organics only. A hierarchical cluster analysis identified key compounds linked to OP, including yet unknown quinones, C_9_H_6_O_6_ (an oxidation product of *o*-xylene), and C_9_H_8_O_4_ (an oxidation product of trimethylbenzene), suggesting secondary products from aromatic precursors. We also found phthalic acid, a tracer for biomass burning and vehicular exhaust, and nitrosalicylic acid, an OP-enhancing compound. A volume-normalized OP of up to 4 nmol DTT min^–1^ m^–3^ was observed after metal removal, underscoring the importance of stable organic compounds and isolating metals to understand their relationship with OP in ambient particulate matter.

## Graphical abstract

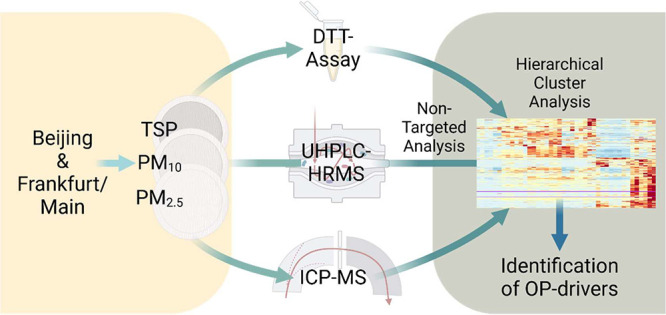

## 1 Introduction

Air pollution and health, which is inevitably linked to aerosols, have been a topic of concern ever since human activity began to outpace natural processes. Major health concerns arise due to a daily exposure, leading to adverse health effects.^1^ Recent studies show a strong correlation between mortality and particulate matter (PM) exposure,^2–4^ suggesting a global excess mortality rate of 8.8 million caused by air pollution.^3^

In general, ambient PM can be formed by various processes, leading to different chemical composition, size, and therefore various health effects.^5,6^ Notable, fine particles with an aerodynamic diameter less than 2.5 μm (PM_2.5_) can enter deeply into the respiratory system and cause severe health issues such as cardiovascular diseases,^1,7^ lung cancer, and lower respiratory tract infections.^3^ A major fraction of fine PM is classified as organic matter,^8^ which consists of both primary and secondary organic aerosol (POA and SOA), as well as oxidation products of primary PM species.^9^ Atmospheric organic aerosols hereby occur in high concentrations in urban and remote locations,^9^ where one formation pathway is the atmospheric oxidation of volatile organic compounds (VOCs), eventually involving inorganic trace gases such as NO_*x*_ and SO_2_. Estimations suggest that 10,000 to 100,000 different organic compounds in the atmosphere undergo various reactions, leading to the formation of complex atmospheric organic aerosols.^10,11^ Since the overall impact of PM on health still remains highly uncertain,^12,13^ an identification and characterization of specific chemical compounds is essential to understand the origin and formation pathways of health-relevant compounds.^14^

To further study adverse health effects, the oxidative potential (OP), as one potential proxy for PM toxicity, was established.^15–18^ Increasing evidence supports OP as a better marker for health effects than PM mass concentration^19–22^ and especially water-soluble (WS) organic compounds have gained more attention in regard to OP.^17,23–26^ OP hereby describes the likeliness to induce oxidative stress due to excess reactive oxygen species (ROS) and simultaneous depletion of antioxidants, causing damage to cells, proteins, lipids and DNA.^27,28^ Commonly used assays are the ascorbic acid assay (OP^AA^), the dithiothreitol assay (OP^DTT^), and the glutathione assay (OP^GSH^). These assays differ in their sensitivity to different species that drive OP. While OP^GSH^ is mostly sensitive to metals, OP^AA^ and OP^DTT^ respond to metals as well as organics.^17^ Earlier studies on OP^DTT^ by Charrier and Anastasio^29^ highlight the importance of WS metals and a few organic compounds causing OP, whereas several recent studies emphasize the significant contribution of WS organic compounds.^23–25,30^ This increased interest in WS organic compounds simultaneously led to more questions about sources, the anthropogenic influence, and interactions between different species influencing OP.^31^ To answer these questions, we therefore chose the acellular OP assay using DTT, which is an established method and well-described in the literature. DTT hereby acts as a surrogate for reducing agents, which simulates the effect of chemical compounds on antioxidants. The DTT consumption over time is thus proportional to the concentration of redox-active compounds.^29,32,33^ This assay is particularly sensitive to copper, manganese, and certain organic species, for example, quinones and humic-like substances.^29,31,34^ However, the response to different singular organic compounds and hence the main drivers remain largely unknown. By using the column material Chelex 100 resin (Chelex) for sample pretreatment, WS metals can be removed and the WS OP^DTT^, caused by organic compounds, can be observed separately.^35^

To contrast the effects of different chemical compositions, we analyzed 42 samples from four different locations across Germany and China. To cover different site types that can cause variability in OP,^36^ we selected Beijing as a highly populated and polluted city, Frankfurt Hoechst as an urban/industrial site, the Taunus Observatory as a rural background site, and Frankfurt Riedberg as an urban background site. We also investigated different particle cutoffs for their WS OP^DTT^, the overall chemical composition, as well as the WS metal contribution. Previous research mostly focuses on specific classes of organics, grouped compounds defined by their origin or target compounds, which do not account for the complex organic aerosol present in the atmosphere. Hence, we chose a nontargeted approach to unveil yet unknown compounds, getting us closer to understanding the compounds that drive OP. Here, we analyzed the fraction of WS organic compounds by performing ultrahigh performance liquid chromatography (UHPLC) combined with high-resolution Orbitrap mass spectrometry (HRMS) and quantified WS metals via inductively coupled plasma mass spectrometry (ICP-MS).

To combine the findings of chemical characterization and OP^DTT^ analysis, we applied a hierarchical cluster analysis (HCA) on the merged data set to identify specific compounds and compound groups that are potential chemical drivers of WS OP^DTT^. Our findings therefore contribute to a better understanding of the relationship between WS OP^DTT^ and WS organic compounds, including potential sources, while also pointing out singular compounds that are relevant to WS OP^DTT^.

## 2 Material and Methods

### 2.1 Sample Origin and Collection

In this work, we analyzed ambient air filter samples of the Taunus Observatory at Kleiner Feldberg (TO, 50.2219° N, 8.4468° E), Beijing (40.0426° N, 116.4197° E), Frankfurt Riedberg (GEO, 50.1736° N, 8.6337° E), and Frankfurt Hoechst (HOC, 50.1017° N, 8.5425° E). For each location, seven filters with corresponding filter blanks were selected (SI, Tables S1 and S2). All filter samples were collected on preheated glass fiber filters (MG 160, AHLSTROM MUNKSJÖ), sealed, and stored at −20 °C.

The filter samples from TO, GEO, and HOC were collected using a high-volume sampler (DHA-80, Digitel Switzerland) with a PM_10_ preseparator. An additional collection with a PM_2.5_ preseparator was made at TO.

The Beijing total suspended particle (TSP) filter samples were collected with a high-volume sampler (TH-1000C, Wuhan Tianhong Environmental Protection Industry Co. Ltd.). Additionally, PM_2.5_ samples were collected by a sampler from Leckel GmbH with a PM_2.5_ preseparator.

### 2.2 Sample Extraction

For analysis by (a) UHPLC-HRMS, (b) ICP-MS, and (c) WS OP^DTT^, the samples underwent aqueous extraction. The extraction was carried out by taking (a,b) one punch or (c) several punches resulting in a PM concentration of ∼20 μg mL^–1^ (SI, Table S2). The punches with a diameter of (a) 20 mm, (b) 20 mm for HOC, TO, GEO and 12 mm for Beijing, or (c) 14.2 mm were cut and wetted with (a,b) 200 μL or (c) 5 mL of ultrapure water (MQ, Milli-Q Reference A+, Merck KGaA) and placed on an (a, b) orbital shaker (KS-15, Edmund Bühler GmbH) at 300 rpm or (c) an incubator (12 L Shaking Bath, VWR) at 21 °C and 150 rpm, for 20 min. Subsequently, the solvent was extracted from the filter pieces and filtered using a polytetrafluoroethylene filter (Thermo Fisher Scientific Inc., 0.2 μm pore size). Then, another (a,b) 100 μL or (c) 5 mL of MQ was added to the filter pieces, and the procedure was repeated (SI, Figure S1). The finished extracts of procedures (a) and (b) were stored at −20 °C for later measurement via UHPLC-HRMS (a) or ICP-MS (b). The extracts prepared for the WS OP^DTT^ measurement (c) were measured directly.

### 2.3 UHPLC-HRMS Measurements

We analyzed the extracts using a setup that combines UHPLC (Vanquish Flex) with HRMS (Q Exactive Focus Hybrid-Quadrupole Orbitrap, both Thermo Fisher Scientific Inc.). As an ion source, heated electrospray ionization was chosen, and measurements were made in both negative and positive ion modes. The chromatographic separation was performed on a reversed phase column (C_18_-column, CORTECS T3, 2.7 μm, 3 × 150 mm, Waters) at 40 °C (SI, Figure S2).

We employed the separation according to Thoma et al.^37^ by a gradient elution with MQ and methanol (Optima LC/MS grade, Thermo Fisher Scientific Inc.), both with 0.1% formic acid over 20 min, 5 μL injection volume, and a flow rate of 0.4 mL min^–1^. The spectrum was acquired in the full-scan mode, in a mass range of 70–750 *m*/*z* and a resolution of 70,000 at *m*/*z* 200. The resolution for data-dependent MS^2^ acquisition was set to 17,500 (details in SI, Table S3). To evaluate instrumental performance, the intensity variation (SI, Figure S3) and mass accuracy (SI, Figure S4) of four different compounds were quantified.

### 2.4 ICP-MS Measurements

The extracts were dried, and the residues were dissolved in 1 mL of 2% HNO_3_ (ROTHER, distilled) with 5 ppb indium as an internal standard. The measurements were performed with a high-resolution sector field ICP-MS (Element XR, Thermo Scientific Inc.). A continuous injection of 2 min at a flow rate of 150 μL min^–1^ was chosen. For the measurements of manganese (Mn), iron (Fe), copper (Cu), zinc (Zn), and lead (Pb) at a resolution of 4,000, the isotopes ^55^Mn, ^56^Fe, ^65^Cu, ^66^Zn, and ^208^Pb were selected.

### 2.5 Measurements of Water-Soluble OP^DTT^

A total volume of 10 mL was extracted for the OP^DTT^ measurements, 6 mL of which was rinsed through a packed polypropylene gravity flow column (6 mL with 20 μM frits, Marvelgent) to remove the metals (SI, Figure S5b). The column was prepared based on Wang et al.,^35^ with ∼0.8–0.9 g of Chelex (200–400 mesh particle size, Merck KGaA) and prerinsed with 500 mL of MQ to decrease the effluent pH to 7–8. The OP^DTT^ assay was then carried out based on the method by Cho et al.^32^ and is described here only in brief, with a detailed description in the SI. All steps were applied to both Chelex-treated and untreated extracts (SI, Figure S5a) and performed under light avoidance to exclude effects on the reactants and reaction products.

All samples were prepared in duplicates, and a concentration of 100 μM DTT in the incubation vial (MQ100, Merck KGaA) was used for the assay. During an incubation time of 30 to 45 min at 37 °C and 150 rpm (12 L Shaking Bath, VWR), four aliquots were taken out after equal time steps and quenched with 10% trichloroacetic acid (99%, Merck KGaA). The quantification was performed by adjusting the pH to 8.9 and adding 5,5-dithiobis-2-nitrobenzoic acid (98%, Merck KGaA) to form the chromophore 5-thio-2-nitrobenzoic acid. Subsequently, the optical density was measured at λ_abs_ = 412 nm by a microplate reader (CLARIOstar^Plus^, BMG LABTECH GmbH). The different types of OP mentioned are summarized in Table 1. In addition to the sample measurements, a filter blank, a reagent background, a positive control, and a calibration curve were measured (SI, Figures S6 and S8) for all samples. The positive control using 9,10-phenanthrenquinone as a DTT-active compound resulted in ∼0.7 μM DTT min^–1^ for both treated and untreated samples.

**Table 1:** Overview of the Different OPs Referenced Below

	how it is assessed	compound types it refers to
OP_V_	DTTassay	WS metals and organics
OP_V,Cx_	Chelex treatment, subsequent DTT assay	WS organics
OP_V,metal_	theoretical calculation of OP caused by WS metals	WS metals

### 2.6 Nontargeted Analysis and Group Assignment

For the nontargeted analysis (NTA), FreeStyle 1.8 SP2, Compound Discoverer 3.3 (CD) (both Thermo Fisher Scientific Inc.), and PythonSpyder 5.4.1 (Python Software Foundation) were used to process raw data. As part of NTA, mass spectra containing *m*/*z* and isotopic patterns, MS^2^ spectra containing fragmentation patterns, and the retention time (rt) are used by CD to identify possible compounds and assign a potential molecular formula. The workflow applied, based on Thoma et al.^37^ can be found in detail in the SI. Compounds with a sample-to-blank ratio of less than five are marked as the background. The allowed elements to predict the compositions were bromine (Br), chlorine (Cl), carbon (C), oxygen (O), hydrogen (H), sulfur (S), nitrogen (N), and phosphorus (P). Depending on the molecular formula, each compound was assigned to an elemental composition group (CHO, CHNO, CHNOS, CHOS, CHOP, CHN, and ‘other’). Here, it must be noted that the compound group does not necessarily lead to the conclusion of functional groups. Although, some assumptions can be made based on the polarity mode in which the compounds were detected. By estimating the aromaticity equivalent (Xc) according to Yassine et al.^38^ (SI, eqs 1 and 2), the CHO group was split up into CHOa (aromatic organics) and CHOn (aliphatic organics).

To compare MS^2^ spectra with established libraries and gain information about possible structures, mzCloud (HighChem LLC, 2013–2021) and mzVault 2.3 (Thermo Fisher Scientific Inc.) were used. Hereby, mzVault 2.3 contains the database *Aerosolomics*, which is based on experiments for specific chemical systems and contains information about *m*/*z*, rt, and MS^2^ spectra.^37^

### 2.7 Data Analysis for ICP-MS and WS OP^DTT^

The obtained ICP-MS raw data were corrected by the solvent blank (2% HNO_3_), and the concentrations were calculated by an internal standardization process. The known quantities of the standard (SI, Table S4) can be used to calculate the concentration for each element (SI, eqs 5 and 6). To calculate the loss rate of DTT, the optical density was corrected by the measured reagent background. Furthermore, a calibration curve was used to determine the DTT concentration (SI, Figure S7, eqs 7 and 8). The slope of the resulting curve represents the WS OP^DTT^ in μM DTT min^–1^. All samples were corrected with the corresponding filter blank and normalized. ICP-MS data was normalized according to its corresponding air volume (SI, eqs 9 and 10). In case of OP^DTT^ measurements, normalization was done according to air volume (OP_V_ in nmol DTT min^–1^ m^–3^) and mass (OP_M_ in pmol DTT min^–1^ μg^–1^) (SI, eqs 9–14 and Table S2). To assess the effects of exposure, the OP_V_ represents a good proxy for toxicity^17^ and will be mainly used in this work. All chemical characteristics determined by UHPLC-HRMS and ICP-MS were volume-normalized and blank-corrected and were therefore compared to OP_V_.

### 2.8 Hierarchical Cluster Analysis

The HCA was performed by MATLAB R2022b (The MathWorks Inc.^39^) using the bioinformatics toolbox version 4.16.1. As input, a matrix containing all compounds, both in negative and positive modes with the corresponding intensity for each sample, OP_V_, OP_V_ of the Chelex-treated samples (OP_V,Cx_), and the metal concentration was chosen. For determining the proximity between the observation pairs, the Euclidean distance (SI, eq 3) was used after performing a *z*-transformation (SI eq 4).^40^ For merging different compounds and samples into clusters, the Ward linkage method was used.^40,41^ The clustering was performed on rows (compounds, metals, and OP results) and columns (samples).

## 3 Results and Discussion

### 3.1 WS OP^DTT^ Activity of Different Sample Sites

To investigate the WS OP^DTT^ of WS organic compounds only, as well as in combination with WS metals separately, we compared the OP^DTT^ for each sample without (OP_V_) and with Chelex treatment (OP_V,Cx_). By this approach, we were able to assess potential synergistic or antagonistic effects that WS metals might have on WS organic compounds.^35^ To additionally estimate the theoretical contribution of WS metals to OP, we calculated OP_V,metal_ based on laboratory experiments with WS metals by Charrier and Anastasio (2012).^23,29,42^ Notably, OP_V,metal_ contributes for most samples less than 2% to the measured OP_V_, indicating a major contribution of organic matter to OP_V_ (SI, Table S5).^23,42^ The highest OP_V,metal_ was found for Beijing TSP samples with a contribution of up to 22%, caused by the high concentrations of copper. When comparing the theoretical OP_V,metal_ to the measured reduction in OP_V_ by removing the metals, we found that the theoretical reduction is mostly smaller than the actual reduction in OP_V_. This phenomena underline potential synergistic or antagonistic effects between WS metals and WS organic compounds.^31^ Since the metal removal seems to be dependent on the sample matrix as well as the species, we suspect a general underestimation of OP_V_ reduction due to incomplete metal removal for certain samples (SI, Figure S8 and Table S6). Further measurements show that the organics are not affected by the Chelex treatment (SI, Figure S9). Additionally to be mentioned are the uncertainties derived by the OP^DTT^ method itself, especially when comparing data from different laboratories, which has been thoroughly investigated in a recent intercomparison campaign.^43^

Overall, our measured OP_V_ values fall within the range of other studies that investigated, for example, Beijing (0.8–6.5 nmol DTT min^–1^ m^–3^),^16^ locations in Europe like Athens (3.51 nmol DTT min^–1^ m^–3^)^44^ or Bern (1–4.6 nmol DTT min^–1^ m^–3^),^23^ and Los Angeles (0.4–0.66 nmol DTT min^–1^ m^–3^).^24^ Alongside additional studies investigating the OP_V_ across Asia, North America, and Europe, our measured OP_V_ and OP_V,Cx_ are displayed in Figure 1.^16,23,24,42,44–46^

**Figure 1:**
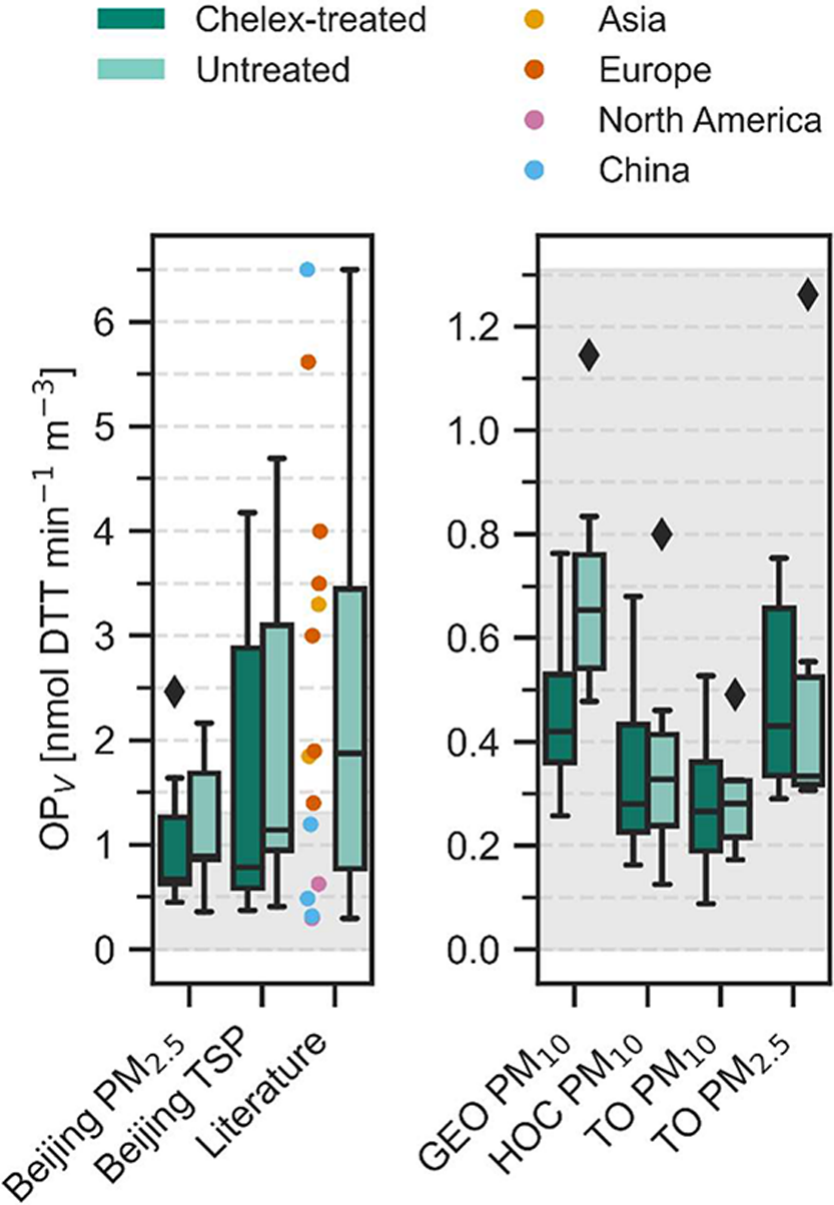
OP_V_ in nmol DTT min^–1^ m^–3^ for the locations Beijing, GEO, HOC, and TO, displayed as a boxplot containing seven samples each (same period for Beijing PM_2.5_ and TSP and same period for GEO, HOC, and TO). Light green represents untreated samples and dark green Chelex-treated samples. The rhombus mark outliers, which are defined as *x* < Q1 – 1.5 · IQR or *x* > Q3 + 1.5 · IQR with IQR = Q3 – Q1. Additionally, values found in the literature are displayed as a boxplot and the corresponding values from various locations in North America, Asia, China, and Europe are marked as colored dots. The according literature is mentioned in the text.

Samples from Beijing and Frankfurt (GEO, HOC, and TO) show a strong difference regarding the range of OP_V_, where samples representing Beijing TSP show the highest, and samples representing TO the lowest OP_V_. For Beijing samples, the OP_V_ ranges from 0.36 to 4.69 nmol DTT min^–1^ m^–3^ and OP_V,Cx_ from 0.37 to 4.17 nmol DTT min^–1^ m^–3^, showing a clear decrease in OP due to removal of WS metals. A comparison of Beijing PM_2.5_ to Beijing TSP highlights that half of the WS OP-active PM in Beijing derives from PM_2.5_, emphasizing the importance of fine PM. Here, sources like dust, traffic, biomass combustion, SOA, and coal combustion are known to contribute significantly to OP derived from PM_2.5_ in Beijing.^47^

For Frankfurt, the OP_V_ ranges from 0.13 to 1.26 nmol DTT min^–1^ m^–3^ and for OP_V,Cx_ ranges from 0.09 to 0.76 nmol DTT min^–1^ m^–3^ and does not differ significantly between locations. Moreover, the removal of WS metals does not change the measured OP_V_ as much as it did in the Beijing samples, pointing toward lower contribution from WS metals. Since PM_2.5_ represents a fraction of PM_10_,^48^ the OP_V_ expected for TO PM_10_ should be higher than for TO PM_2.5_. Here, uncertainties regarding the normalization coming from the high-volume sampler or due to the OP methods’ uncertainty might lead to this incoherence.^49^ Moreover, especially the OP_V_ for TO, representing a rural area, compared to HOC, representing an industrial area, was expected to be significantly lower. However, studies from Dimitriou and Kassomenos show that low polluted areas can be highly affected by air masses coming from regional terrains and also neighboring countries, impacting the local air quality.^50^ During our sampling period, the TO was affected by low altitude air masses coming from the southwest or north, possibly transporting the pollution from cities in these areas and thus leading to an equally high OP (SI, Figures S10 and S11).

To investigate not only the exposure-related effects but also the different reactivities related to the chemical composition, we want to highlight WS OP_M_ (SI, Figure S12), the mass normalized OP, which ranges from 6.35 to 61.66 pmol DTT min^–1^ μg^–1^. Untreated Beijing PM_2.5_ samples show the highest values followed by samples from HOC with 12.81 to 51.25 pmol for untreated and 11.86 to 43.60 pmol DTT min^–1^ μg^–1^ for Chelex-treated extracts. Here, industry-related compounds^51^ seem to have an important OP-reactivity, highlighting the importance of different chemical compositions. To contrast this observation, TSP samples from Beijing have a seemingly low WS OP_M_ of 7.5 to 17.8 pmol DTT min^–1^ μg^–1^ that can be explained by a large contribution of mass with low OP-reactivity, such as dust and insoluble PM, to the atmospheric aerosol. Here, the different PM size fractions show a reverse correlation between particle size and the contribution to WS OP_M_.

### 3.2 Water-Soluble Metal Concentration in Beijing and Frankfurt PM

To understand the contribution of WS metals to WS OP^DTT^ and highlight the differences in chemical composition at each site, we quantified the WS fraction of Mn, Cu, Fe, Zn, and Pb by ICP-MS, as some of the most relevant metals.^29,42^

The highest metal concentrations are found in Beijing TSP with concentrations about 50 times higher (∼180 ng m^–3^) than those in Frankfurt (∼3 ng m^–3^) (Figure 2A). The results in Figure 2A show a metal concentration in Beijing PM_2.5_ of ∼23 ng m^–3^, which is about ten times higher. The contents of WS metals are within the same ranges as shown by Fang et al., who sampled different areas in the South-East United States (US).^52^ The urban US sites are comparable to the concentrations in Beijing PM_2.5_, whereas the concentrations in Frankfurt are much lower than those in rural areas in the United States. In earlier studies, high concentrations of copper and iron were attributed to traffic related sources,^52^ brake or tire wear, mineral dust, and biomass burning.^53–56^ The strong concentration differences are thus linked to the different sources that are site specific. An example would be more traffic at the Beijing or Hoechst sampling site compared to that at the Taunus Observatory.

**Figure 2:**
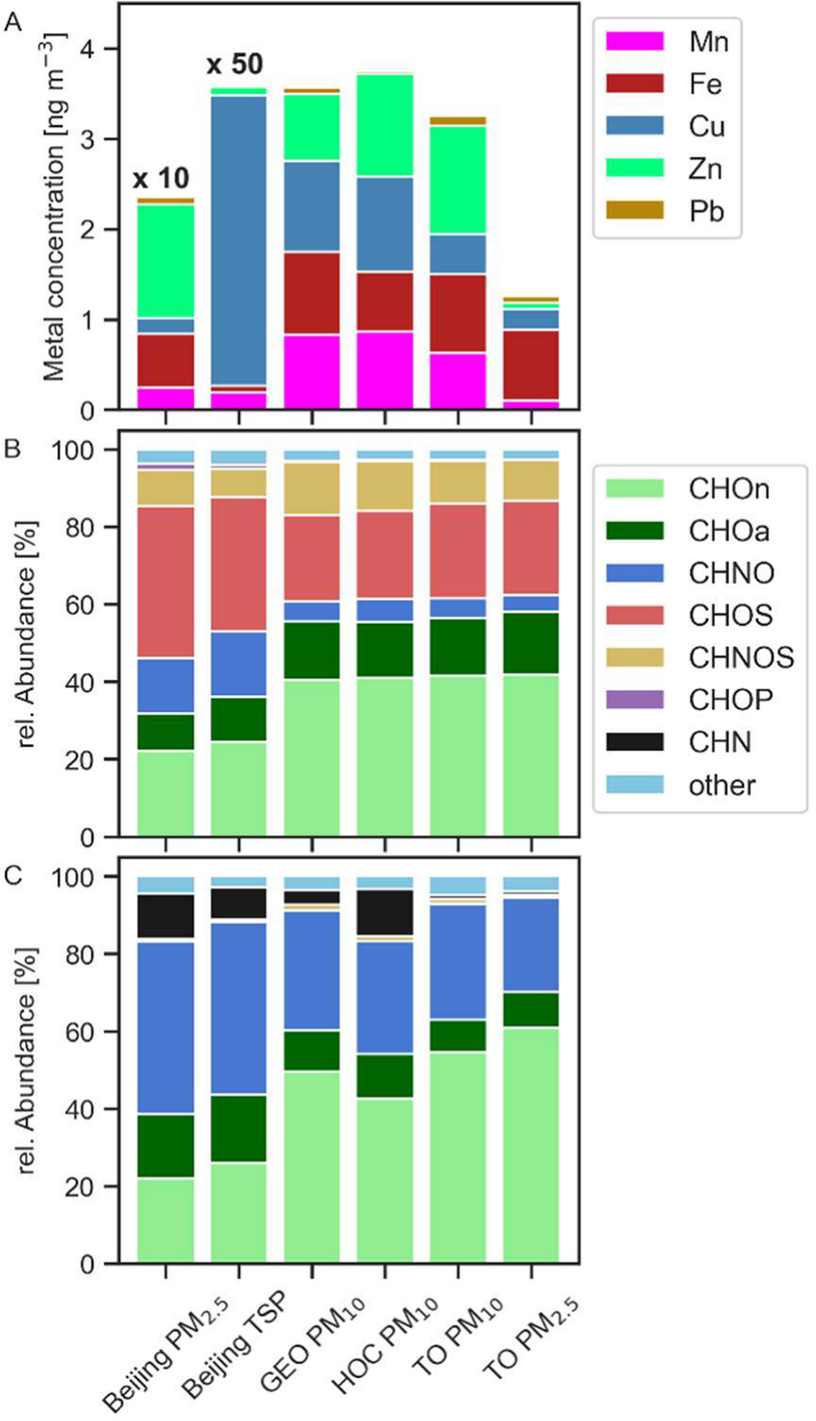
A: Mean concentrations of WS metals (Mn, Cu, Fe, Pb, Zn) in ng m^–3^ and mean relative abundances for the organic compound groups (CHOa, CHOn, CHOS, CHN, CHNOS, CHOP, CHNO, and others) in %, measured in B: negative ionization mode or C: positive ionization mode, for the sampling sites Beijing, GEO, HOC, and TO. The metal concentration, given on the *y*-axis, was divided by 10 for Beijing PM_2.5_ or 50 for Beijing TSP. We identified TOF 2 (SI, Table S5) as an outlier and removed this sample from the data displayed.

The measurements show that all Frankfurt samples (GEO, HOC, and TO) contain similar amounts of WS metals, ranging from 0.09 to 1.77 ng m^–3^ for each species. Looking at Mn, TO PM_2.5_ has a notable low concentration (0.09 ng m^–3^) compared to Frankfurt PM_10_ (0.63–0.86 ng m^–3^), implying that Mn is mostly in the coarse fraction. When comparing the different particle sizes and samples (HOC, GEO, TO PM_10_ vs TO PM_2.5_), iron is the only metal that shows no prominent change in concentration, showing that it is predominantly present in the fine fraction. The metal content and especially the concentration of copper (Beijing PM_2.5_: 1.7 ng m^–3^ vs TSP: ∼150 ng m^–3^) are reflected in the OP_V_ (Figure 1), emphasizing the different contribution of metals to OP.

### 3.3 Relative Abundance of WS Organic Compound Groups in Beijing and Frankfurt PM

To get a more comprehensive picture of the variation of chemical characteristics at the different measurement sites, the fraction of WS organic compounds is split up into chemical groups. Here, the intensities, represented as relative abundance, are not directly linked to concentrations due to compound-specific ionization efficiency.^57^

The most abundant chemical group in Beijing samples is CHOS (measured in the negative ionization mode, Figure 2B) (∼40%), almost twice as abundant compared to samples from Frankfurt (∼23%). CHOS compounds mainly consist of organosulfates (OS)^58^ and can be tracers for biogenic SOA formation and oxidation of anthropogenic VOC, both under acidic conditions.^59^ The presence of OS is likely to enhance PM and contributes to the negative effect on human health.^1–4,60^ Another important group in all sampling sites is CHO compounds, split into oxidized aliphatics (CHOn), which contribute to more than half, and oxidized aromatics (CHOa). Here, samples from Frankfurt show higher relative abundances in general (∼40% CHOn, ∼15% CHOa) compared to Beijing (∼22% CHOn, ∼10% CHOa). Notably, the fraction of CHOa in CHO is higher in Beijing, which likely contains functionalized polycyclic aromatic hydrocarbons (PAH) and quinones that are contributing to OP by generating ROS.^61,62^

For measurements in the positive mode (Figure 2C), samples from HOC show a high relative abundance of CHN compounds with 12.1% compared to GEO (3.6%) and TO (<1%), which likely occur from amines, as a dominant anthropogenic pollutant.^63^ For Beijing, CHNO compounds show the highest relative abundance (∼45%), which is the second most abundant for Frankfurt samples (∼30%). The findings for CHOa and CHOn are qualitatively in agreement with the measurements in the negative mode. The compounds assigned to ‘other’ show an abundance of ∼3% for both measurement modes for all sites. CHOP compounds mark the lowest abundance, with <1% for positive and ∼1% for negative modes. The exact intensities and metal concentrations for each sample are displayed in SI, Figure S13.

In general, we observed a broad chemical spectrum when comparing the sampling sites in Beijing and the sampling sites in Frankfurt. Here, all sampling sites in Frankfurt apart from HOC show a similar chemical composition, in contrast to Beijing samples, which differ significantly in both particle mass and chemical composition.

### 3.4 Spearman Correlation of WS Compound Groups and Metals with WS OP^DTT^

To describe the relationship between compound groups of WS organic compounds, WS metals, and WS OP^DTT^, and gain a deeper understanding of potential chemical drivers, we used the Spearman correlation as a measure of association between data.^64^ Here, it has to be noted that the strength of correlation does not necessarily translate to the DTT reactivity of the respective group. The correlation rather indicates dependencies of OP^DTT^ regarding different compound groups. For the organics, no significant changes due to the Chelex treatment were confirmed by explicit measurements (SI, Figure S9); therefore, we used the initial intensities for correlations with OP_V,Cx_. Previous studies found strong correlations (*r*_s_) between OP and WS organic compounds and WS metals.^24,65,66^ Figure 3 displays the correlation of OP_V_ for untreated and Chelex-treated samples, with the different WS organic compound groups in positive and negative modes (CHOa, CHOn, CHNO, CHOS, CHNOS, CHOP, CHN, and others) as well as the WS metals (Mn, Fe, Cu, Zn, and Pb). In general, a correlation does not ensure a causal relationship between two variables,^64^ hence our approach is a first step on validating the relevance of certain compound groups. In this study, we consider a correlation coefficient *r*_s_ > 0.50 to indicate a strong correlation,^64^ which is true for Mn, Cu, Fe, CHOS_neg_, CHOa_neg_, CHOa_pos_, other_neg_, CHNO_neg_, and CHNO_pos_.

**Figure 3:**
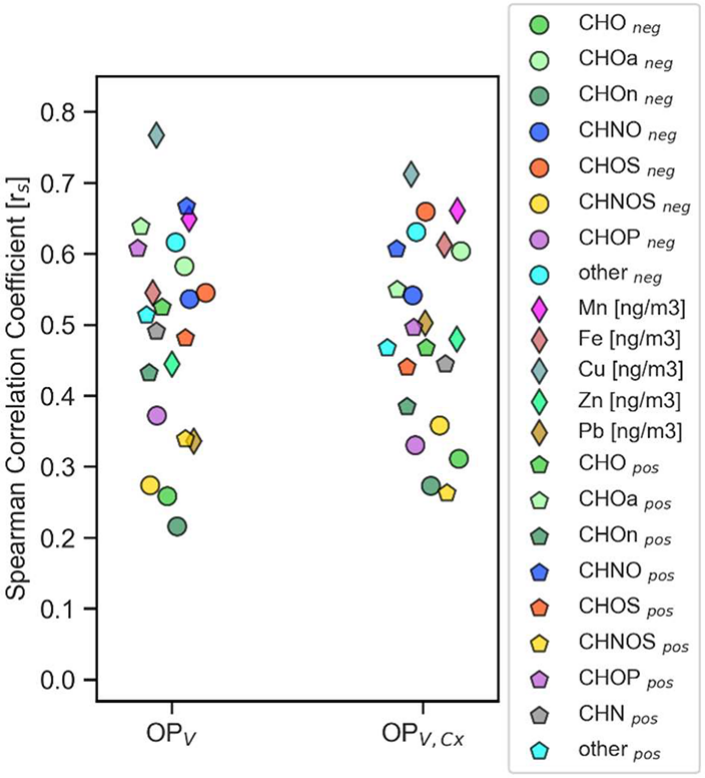
Spearman correlation of WS metals (Mn, Fe, Cu, Zn, and Pb) and WS organic compound groups in negative and positive modes (CHOa, CHOn, CHNO, CHOS, CHNOS, CHOP, CHN, and others) with WS OP^DTT^ derived from untreated samples OP_V_ or Chelex-treated samples OP_V,Cx_.

The correlations of OP_V_ with WS Cu and Fe are stronger than correlations found by Chen et al. (Cu: *r*_s_ = 0.48, Fe: *r*_s_ = 0.19),^65^ which emphasizes the complexity of assigning OP contributions. Mn shows the second-strongest correlation for WS metals (*r*_s_ = 0.64), which is consistent with studies stating Cu and Mn being the most OP^DTT^-reactive metals.^29,67^ Other strong correlations besides Mn, Cu, and Fe occur for CHOa, CHOS_neg_, CHNO_neg_, CHNO_pos_, and other_neg_. These are in general lower than the correlations found by Chen et al. for WS organic carbon (*r*_s_ = 0.85).^65^ The correlation of OP_V_ with CHNO and CHOS indicates potential health risks due to their large contribution to SOA formation.^68–70^ Additional inorganic components of PM such as sulfate and nitrate are reported to cause a potential increase in the bioavailability of other toxic counterions such as transition metals, which can lead to an increase of WS OP.^13^

Weak correlations were observed for Pb, CHNOS_neg, pos_, CHOn_neg_, and CHO_neg_. Here, we emphasize the importance of distinguishing between aromatic (CHOa) and aliphatic CHO (CHOn), since there is no significant correlation between OP^DTT^ and CHO_neg_ or CHOn, but a strong correlation with CHOa for both polarities.

In general, the comparison between OP_V_ and OP_V,Cx_ shows that the overall correlation trend is alike, with only minor changes in *r*_s_. This is due to a similar behavior in variation of OP_V_ to OP_V,Cx_ throughout the samples (SI, Figure S12), although the absolute WS metal-driven OP^DTT^ is reduced after Chelex treatment (SI, Figures S12 and S14), which still can result in a similar Spearman coefficient.

### 3.5 Identifying WS OP^DTT^ Correlated Compounds by Applying HCA

To observe not only the correlation between WS OP^DTT^ and elemental composition groups but also for single compounds, we applied HCA in order to reduce the complexity of the matrix. Hence, observations with similar behavior are clustered together based on the similarity between each pair of objects.^71^ Here, some compounds that do not drive OP^DTT^ can be of the same origin as OP^DTT^-drivers and thus appear in the same cluster; therefore, the individual compounds and effects on OP^DTT^ need to be further studied. Although HCA is based on correlations and cannot ensure causality, a group of compounds closely related to OP^DTT^ may represent potential OP^DTT^-drivers. The stepwise process of fusing the data into clusters is displayed in a dendrogram (SI, Figure S15).^72^

Figure 4 shows the individual organic compounds that are closely linked to the OP^DTT^ observations and, thus, they appear in the same cluster (marked in purple in SI, Figure S15) as OP^DTT^ for Chelex-treated as well as untreated samples. These compounds are likely drivers of OP themselves, transformation products of drivers, or source related compounds, while the remaining compounds are grayed out and indicate no direct link to OP, though it cannot be excluded. To declare the certainty for our compound identification, we used the confidence levels established by Schymanski et al.^73^

**Figure 4:**
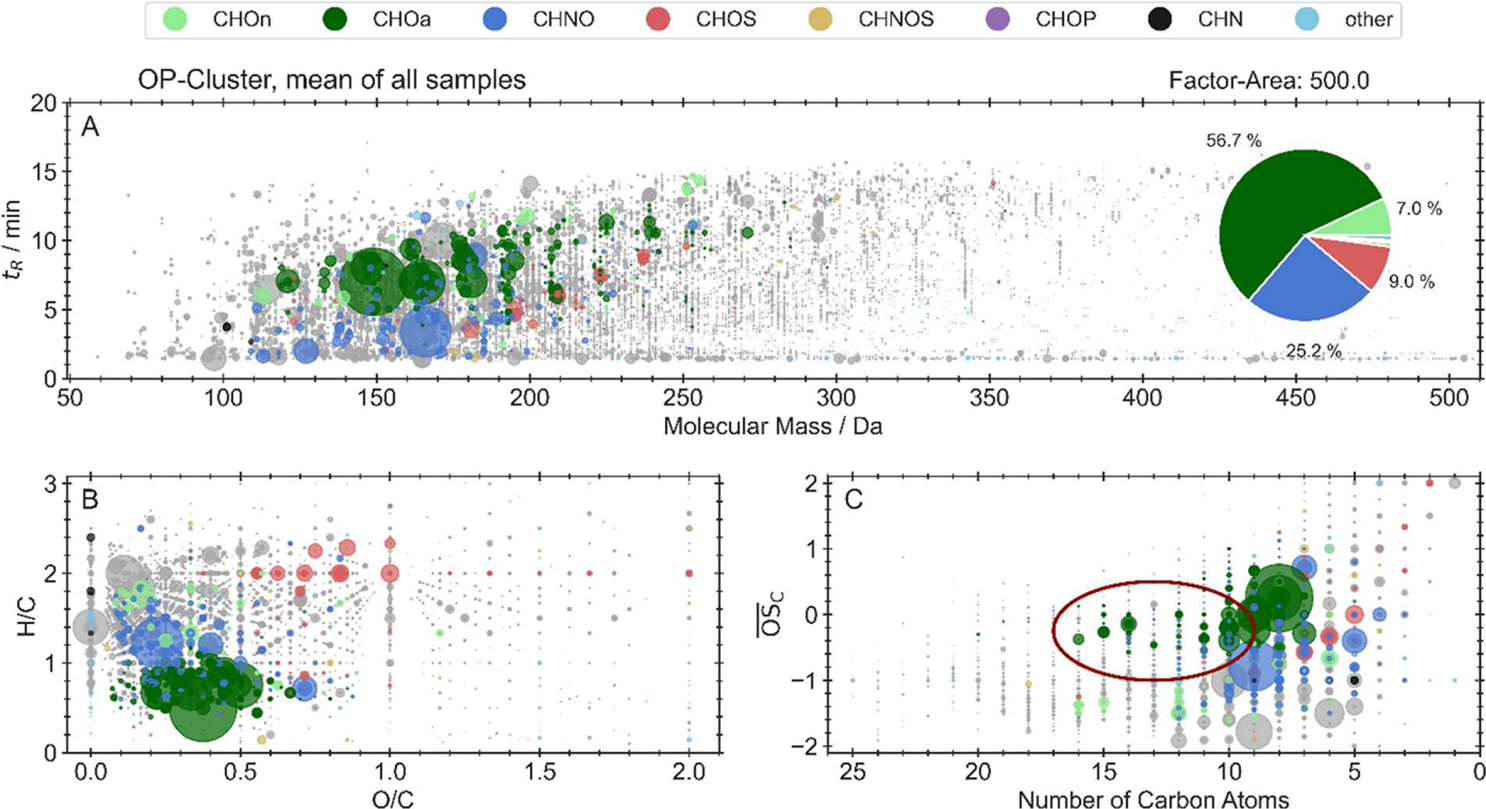
Molecular fingerprint of the OP^DTT^-cluster, representing the mean intensity of all compounds of all samples (measured in negative and positive modes). A: *m*/*z* vs rt. B: Van Krevelen diagram (H/C to O/C). C: Kroll plot ( ${\overset{-}{\mathrm{OS}}}_{c}$ vs number of carbon atoms). Circle diameter represents the relative intensity; gray compounds are not within the OP-cluster; and the red ellipse represents the potential quinones.

The compounds linked to OP^DTT^ are displayed in A: *m*/*z* vs rt, B: the Van Krevelen diagram, displaying the O/C and H/C ratios, and C: the Kroll Plot, which displays the number of carbon atoms vs the average oxidation state of the carbon atom ( ${\overset{-}{\mathrm{OS}}}_{c}$ ). The compounds that contribute to the OP-cluster are mainly CHOa (57.3%) and CHON (25.2%), followed by CHOS (9%) and CHOn (6.4%), which is consistent with the correlations shown in Figure 3.

By HCA, we were able to narrow 12,172 compounds found by NTA down to 642 that are closely linked to WS OP^DTT^. The identified compounds show a range of 110 to 270 Da (Figure 4A), which is a narrow range compared to the remaining compounds that range from 70 to 370 Da.

The rt ranges from 2 to 15 min, covering a wide range of polarities from early eluting polar compounds to late eluting unpolar compounds.^74^ In general, the most abundant compound group within the OP-cluster CHOa contains compounds that we identified using the *Aerosolomics* database.^37^ Here, we identified phthalic acid (C_8_H_6_O_4_, *m*/*z*: 166.026, rt: 6.9 min, level 1) with concentrations reaching 86.50 ng m^–3^ in Beijing samples (SI, Figure S19). Phthalic acid, an oxidation product of highly DTT-active compounds such as naphthalene or other PAHs,^75–77^ is reported as a tracer for emissions from biomass burning, vehicular exhaust, and fossil fuel combustion, indicating OP-relevant sources, since it is not OP-active itself (SI, Figure S20).^78–80^ Moreover, C_9_H_6_O_6_ (*m*/*z*: 210.016, rt: 6.4 min, level 2) was identified as an oxidation product of *o*-xylene and C_9_H_8_O_4_ (*m*/*z*: 180.042, rt: 8.5 min, level 2) as an oxidation product of trimethylbenzene, both marking anthropogenic VOCs.^37^ Here, photooxidation of VOCs can facilitate the formation of oxidizing-active compounds, resulting in an increase in OP and underscoring the importance of secondary formation of OP-active compounds.^81^

Additionally, the most abundant compound groups CHOa and CHNO fall into the region of H/C < 1.2 in the Van Krevelen diagram (Figure 4B), emphasizing an aromatic character.^37^ Hereby, the appearing CHOa compounds show a carbon atom number between 10 and 16 (Figure 4C), indicating the oxidation of larger VOC precursors such as anthracene, phenanthrene (C_14_), or alkylated naphthalene. These compounds additionally show an ${\overset{-}{\mathrm{OS}}}_{c}$ between 0 and −0.2, which applies for quinones. In this regard, we were indeed able to identify C_14_H_8_O_2_ (level 2) as an isomer of anthraquinone by comparing MS^2^ spectra. Since the rt does not match to the standard, we attributed the tentative candidate structure of the 1,2-isomer, which elutes earlier than anthraquinone.^82^ The diagnostic fragments in the MS^2^ spectra are *m*/*z*: 181 (−CO) and 153 (− 2 x CO) (SI, Figure S16). Other comparisons with standards (SI, Figures S17 and S18) underline the presence of unknown quinones, which need to be further identified. Quinones are known to exist in significant concentrations in ambient PM, derived largely from biomass burning and traffic,^83^ and were studied by many researchers with respect to their individual OP.^29,84–86^ Supported by additional experiments, quinones vary significantly in their OP^DTT^-activity as well as detectability via HESI-HRMS (SI, Figure S19).^29^ Due to varying ionization efficiency, semi quantification for our samples ranges from 0.11 to 817 ng m^–3^. Here, we discovered yet unidentified quinones using NTA that might have significant concentrations in the ambient as well as effects on WS OP^DTT^ and need to be studied further. CHNO appears moreover in the region 1.2 ≤ H/C ≤ 1.8, which represents biogenic SOA in the presence of NO_*x*_,^78^ including oxidation products of monoterpenes (e.g., C_10_H_16_),^87,88^ as well as small aromatics such as benzene, toluene, ethylbenzene, or xylene (BTEX). They are indicated by a number of carbon atoms between 5 and 10 and are reported as emissions from traffic and coal burning in the north of China.^89,90^ Especially nitrogen-containing aromatic compounds are known to promote electron transfer due to the N atom acting as a hydrogen-bonding acceptor. Here, we identified C_7_H_5_NO_5_, C_9_H_11_NO_2_, and C_5_H_6_N_2_O_2_ (all level 3) as the most abundant compounds with C_7_H_5_NO_5_ being identified as nitrosalicylic acid with up to 4.20 ng m^–3^ in Beijing samples, which is a tracer for biomass burning and the photochemically oxidation of toluene in the presence of NO_*x*_.^87,91–94^ Though nitroaromatics cannot undergo redox cycling themselves, they act as a catalyst for the redox process of quinones and thus enhance OP, which makes them an important contributor.^81^ Nitrosalicylic acid, in particular, showed the OP^DTT^-activity enhancing effect when present with quinones such as 1,2-naphtoquinone or phenanthrenquinone (SI, Figure S21), suggesting synergistic effects on OP while present with the unidentified quinones. The third most abundant compound group in the OP-cluster CHOS mostly falls within H/C ≥ 2, indicating a saturated aliphatic character likely originating from the combustion of sulfur containing fossil fuel like coal.^95^ Here, C_7_H_16_O_6_S, C_6_H_12_O_5_S, and C_5_H_10_O_5_S (all level 3) showed the highest intensities, whereas C_6_H_12_O_5_S and C_5_H_10_O_5_S show the homologous series of saturated aliphatic OS (C_*n*_H_2*n*_O_5_S), derived from fossil fuel emissions and reactions with SO_2_.^79,87,96,97^ In general, OS was found to reach 0.4 ng m^–3^ in our samples. Further experiments showed no significant OP^DTT^-activity from aliphatic organosulfates (SI, Figure S20); hence, they act as a source marker, emphasizing emissions due to fossil fuel combustion as one of the main sources contributing to OP.^17,36,98^ Additionally, compounds in the lower ${\overset{-}{\mathrm{OS}}}_{c}$ range, partly attributable to CHNO or CHOn, indicate emissions from traffic related sources.^88^ Here, we identified C_8_H_6_O_5_, another photooxidation product of naphthalene (level 2), as well as larger alkanes like C_12_H_22_O_2_, C_15_H_26_O_3_, or C_12_H_20_O_2_ (all level 3) that have not been reported yet in regard to OP. This compound group of WS organic compounds in general is linked to high OP by earlier studies by Fang et al. and Yanosky et al., including mechanical (brake/tire wear) and combustion emissions.^99,100^

In addition to the clustering of compounds, to identify OP-related species, we identified sample clusters as Beijing high polluted (hp), Beijing low polluted (lp), and Frankfurt C1 and Frankfurt C2 (SI, Figure S22) that point out the differences between the locations. Especially with regard to OP, the contribution of different compound groups is crucial for the measured OP^DTT^ and contributes to explaining the observed differences. Here, the comparison between Beijing and Frankfurt shows a higher abundance of CHOS in Beijing, which is redundant with the findings in Figure 2, and higher CHOa content in Frankfurt. The different Frankfurt clusters, containing different samples, emphasize the chemical variety of compounds, even within one sampling site. Here, C1 contains samples that show high abundances of CHOa and C2 contains samples with a higher abundance of CHNO compounds.

The differences between Beijing hp and lp derive from the different time periods of sample collection (SI, Figure S22), possibly reflecting pollution events. Beijing hp contains samples from ninth, 10th, and 15th of March 2022, whereas Beijing lp covers the earlier period from the second until seventh of March 2022. Here, air masses from high altitudes are potentially bringing clean air downward (SI, Figure S23), leading to lp and air masses coming from low-level transport of pollution (SI, Figure S24), leading to hp.

By applying HCA to a data set derived by NTA, we were able to distinguish between compounds that closely relate to OP^DTT^ and compounds that seem to have less correlation to OP^DTT^. By that approach, we reduced the complexity of ambient aerosols, striving to gain more understanding of yet unknown and unreported compounds within PM that drive OP^DTT^. Here, we discovered yet unidentified quinones and compounds along this cluster that open up new research questions regarding their origin and formation pathways. To improve future regulations on air quality and mitigate harmful emissions, we propose monitoring targeted compounds like nitrosalicylic acid, which enhances OP, phthalic acid, a tracer for traffic emissions and biomass burning, or various organosulfates that act as markers for fossil fuel emissions. These targeted standards add to air quality monitoring alongside PM_10_ or PM_2.5_ by either indicating sources that are likely to cause OP or monitoring OP-active compounds itself, which is missing in current health guidelines. Routine measurements of air quality would also benefit from a semi-targeted approach to capture quinone-like structures and thus bridge suspected target screening with a group of OP-relevant compounds that have so far been neglected. To identify and specify sources and emitters as well as improve OP-prediction by models or tools using artificial intelligence, we propose NTA and thus large chemical information data sets.

## 4 Limitations and Implications

The complete representation of the chemical composition is limited by sample collection and the UHPLC-HRMS offline technique. Here, the loss of short-lived species, reactions occurring on the filter, and compounds that are nonionizable or hard to ionize are factors that need to be considered.^101–104^ Additional measurements with a gas chromatography MS system would therefore generate a more comprehensive picture.^105^

The DTT assay measures endogenous ROS rather than the usually very short-lived exogenous ROS.^17^ However, recent studies show that short-lived compounds can also strongly affect OP^DTT^.^43^ The offline analysis of OP^DTT^ therefore represents only the stable fraction of OP^DTT^-active compounds. Moreover, components such as endotoxins or water-insoluble compounds are excluded from this study, which generally play an important role in deriving adverse health effects of PM.^26,106^

Additionally, the effects of metal–organic mixtures on the WS OP are not necessarily linear. Depending on the metal content, antagonistic or synergistic effects can be observed, which then either lead to a lower or higher WS OP.^31,107,108^ Due to these complex effects, the actual exposure effect is not fully represented when removing the metals.

Nevertheless, NTA, isolating WS organic compounds from WS metals, and OP^DTT^ measurements combined with HCA are the first important steps to gain a more comprehensive understanding of chemical drivers of WS OP. Here, OP-prediction models would benefit from more nontargeted based data sets, investigating the complex relationships between various compounds and the resulting OP. Especially in the context of ambient aerosols, which are complex and heterogeneous mixtures, the individual compounds that drive health effects continue to be little studied.^109^ In this study, we identified strongly correlated compound groups such as CHOS and CHOa as well as singular compounds like nitrosalicylic acid, a tracer for biomass burning, phthalic acid, a tracer for traffic emissions and biomass burning, and yet unresolved quinones that potentially drive OP^DTT^. Here, further isolation of compounds and quantification are needed to understand their individual contributions, as well as comparisons to online methods, as presented by Utinger et al.,^110^ or other acellular methods. To study potential sources and compensate for the lack of authentic standards in the context of atmospheric measurements, more oxidation flow reactor experiments according to the *Aerosolomics* approach, such as presented by Thoma et al.,^37^ should be carried out.

By showing the power of NTA, combined with a clustering approach that highlights WS organic compounds that contribute to OP, we encourage more research in that field to create a large database to further predict OP and improve human health as a joint effort.

## Supplementary Material



## Notes

Sample overview and detailed information on the sampling; extraction method and instrument setup schemes; additional measurements of measurement stability and mass accuracy; the detailed OP^DTT^ protocol; detailed information on CD-workflows; equations on aromaticity equivalent; equations used for the HCA; DTT calibration curves; detailed explanation on normalization; further measurements of metal removal efficiency and effects; backward trajectories for Taunus Observatory; parameter overview; detailed HCA output; additional MS^2^ spectra for targeted compounds; measurements of the nitrosalicylic enhancing effect; sample cluster molecular fingerprints; and backward trajectories for Beijing (PDF)

## Abbreviations

CD: Compound Discoverer
Chelex: Chelex 100
DTT: dithiothreitol
GEO: Frankfurt Riedberg
HCA: hierarchical cluster analysis
HOC: Frankfurt Hoechst
hp: high pollution
HRMS: high-resolution mass spectrometry
ICP: inductively coupled plasma
lp: low pollution
*m*/*z*: mass to charge ratio
MQ: ultrapure water Milli-Q
MS: mass spectrometry
NTA: nontargeted analysis
OP: oxidative potential
OS: organosulfate
${\overset{-}{\mathrm{OS}}}_{c}$: average oxidation state of the carbon atom
PAH: polycyclic aromatic hydrocarbons
PM: particulate matter
ROS: reactive oxygen species
rt: retention time
SI: supporting information
SOA: secondary organic aerosols
TO: Taunus Observatory
TSP: total suspended particle
UHPLC: ultrahigh performance liquid chromatography
VOC: volatile organic compounds
WS: water-soluble
Xc: aromaticity equivalent
