# Supplementary material for: Nontargeted Analysis Reveals Organic Compounds That Drive Oxidative Potential in Ambient Particulate Matter
Source: Environ Sci Technol. 2026 Feb 23;60(9):7224–36. doi: 10.1021/acs.est.5c07847 (PMC12980843; doi:10.1021/acs.est.5c07847)
Supplement: Supplementary file 1 [file es5c07847_si_001.pdf]

Supporting Material to:

Nontargeted Analysis Reveals Organic Compounds that Drive Oxidative Potential in Ambient Particulate Matter

Anna Breuninger [†,⊥,‡], *Alexander Schmidt* [⊥,§], *Florian Ungeheuer* [†], *Lingli Zhou* [∥], *Jialiang Ma* [†], *Sarah S. Steimer* [‡,*] *and Alexander L. Vogel* [†,⊥,*]

[†] Institute for Atmospheric and Environmental Sciences, Goethe University Frankfurt, 60438 Frankfurt am Main, Germany

[⊥] Frankfurt Isotope and Element Research Center (FIERCE), Goethe University Frankfurt, 60438 Frankfurt am Main, Germany

[‡] Department of Environmental Sciences, Stockholm University, Stockholm, 11419, Sweden

[§] Institute for Geosciences, Goethe University Frankfurt, 60438 Frankfurt am Main, Germany

[∥] South China Institute of Environmental Sciences, Ministry of Ecology and Environment, Guangzhou, 510655, China

*(S.S.S.) E-Mail: sarah.steimer@aces.su.se

*(A.L.V.) E-Mail: vogel@iau.uni-frankfurt.de

Document Contains:

70 Pages

24 Figures

6 Tables

Content of SI:

I, Table S1, Sample overview……S4

II, Table S2, Overview Detailed Information of the Analyzed Samples……S6

III, Figure S1, Extraction Method……S8

IV, Figure S2, UHPLC-HRMS Setup……S9

V, Table S3, UHPLC-HRMS Method……S10

VI, Figure S3, Quality Control of Measurement Stability……S11

VII, Figure S4, Mass Accuracy……S14

VIII, Figure S5, OP Measurements……S15

IX, OP^{DTT}-Protocol, detailed……S16

X, Figure S6, Positive Control……S18

XI, Compound Discoverer Workflow, negative mode……S19

XII, Compound Discoverer Workflow, positive mode……S31

XIII, Aromaticity Equivalent according to Yassine et al……S43

XIV, z-Transformation and Euclidean Distance……S44

XV, Figure S7, DTT Calibration Curve……S45

XVI, Calculation of the Metal Concentration……S46

XVII, Table S4, Standard Solution for ICP-MS……S47

XVIII, Normalization according to Air Volume……S48

XIX, OP Mass and Volume Normalization……S49

XX, Table S5, Metal Concentrations and theoretical $OP_{V,metal}$……………………………..………S50

XXI,Figure S8, Metal-Removal by Chelex Resin …….………………..………………..…S52

XXII,Table S6, Metal-Removal Efficiency by the Chelex Resin……………….………....S53

XXIII,Figure S9, Effect on Organic Compounds by Chelex Resin……………..……...….S54

XXIV,Figure S10, Backwards Trajectory TO, 29th of April to 7th of May 2022……....…S55

XXV,Figure S11, Backwards Trajectory TO, 7th to 15th of May 2022……………….……..S56

XXVI,Figure S12, Measured OP_M for each Location ………………………………..….…S57

XXVII, Figure S13, Overview of all Measured Parameters for each Sample …..……..…...S58

XXVIII, Figure S14, Copper-Removal by Chelex Resin ……………………………..….…S59

XXIX, Figure S15, HCA………………………………..…………………………...…S60

XXX, Figure S16, MS^2-Spectra of 9,10-Phenanthrenquinone……………….…………………..S61

XXXI, Figure S17, MS^2-Spectra of Anthraquinone…………………………..……………S62

XXXII, Figure S18, MS^2-Spectra of 1,2-Naphtoquinone……………………………..……….S63

XXXIII, Figure S19, Calibration Curves …………………………...…………………S64

XXXIV, Figure S20, OP-Activity of Targeted Compounds …………………..……..……S65

XXXV, Figure S21, Effect of Nitrosalicylic Acid on OP-activity of Quinones ………..….S66

XXXVI, Figure S22, Molecular Fingerprints for Sample Clusters…………………………S67

XXXVII, Figure S23, Backwards Trajectory Beijing, 27th of February to 7th of March 2022………………………………………………………………………………S68

XXXVIII, Figure S24, Backwards Trajectory Beijing, 7th to 15th of March 2022……..….S69

I, Table S1, Sample overview

Table S1. Overview of the analyzed samples, including information about the date, location and the corresponding blank. For Beijing, D means day and N means night.

Sample Name	Date	Location	Corresponding Blank
Beij 25.1	2022.03.02D	Beijing	Beij 25.B
Beij 25.2	2022.03.02N	Beijing	Beij 25.BN
Beij 25.3	2022.03.05D	Beijing	Beij 25.B
Beij 25.4	2022.03.07D	Beijing	Beij 25.B
Beij 25.5	2022.03.09D	Beijing	Beij 25.B
Beij 25.6	2022.03.10D	Beijing	Beij 25.B
Beij 25.7	2022.03.15N	Beijing	Beij 25.BN
Beij T.1	2022.03.02D	Beijing	Beij T.B
Beij T.2	2022.03.02N	Beijing	Beij T.BN
Beij T.3	2022.03.05D	Beijing	Beij T.B
Beij T.4	2022.03.07D	Beijing	Beij T.B
Beij T.5	2022.03.09D	Beijing	Beij T.B
Beij T.6	2022.03.10D	Beijing	Beij T.B
Beij T.7	2022.03.15N	Beijing	Beij T.BN
HOC 009	2022.05.03	Frankfurt Hoechst	HOC 001
HOC 012	2022.05.06	Frankfurt Hoechst	HOC 010
HOC 014	2022.05.09	Frankfurt Hoechst	HOC 010
HOC 015	2022.05.11	Frankfurt Hoechst	HOC 010
HOC 016	2022.05.12	Frankfurt Hoechst	HOC 010
HOC 018	2022.05.14	Frankfurt Hoechst	HOC 017

HOC 019	2022.05.15	Frankfurt Hoechst	HOC 017
TOF 1 (TOF 792 + TOF 793)	2022.05.03	Taunus Observatory	TOF 57
TOF 2 (TOF 798 + TOF 799)	2022.05.06	Taunus Observatory	TOF 57
TOF 3 (TOF 804 + TOF 805)	2022.05.09	Taunus Observatory	TOF 58
TOF 4 (TOF 808 + TOF 809)	2022.05.11	Taunus Observatory	TOF 58
TOF 5 (TOF 810 + TOF 811)	2022.05.12	Taunus Observatory	TOF 58
TOF 6 (TOF 814 + TOF 815)	2022.05.14	Taunus Observatory	TOF 59
TOF 7 (TOF 816 + TOF 817)	2022.05.15	Taunus Observatory	TOF 59
TO 045	2022.05.03	Taunus Observatory	TO 040
TO 049	2022.05.06	Taunus Observatory	TO 048
TO 052	2022.05.09	Taunus Observatory	TO 048
TO 054	2022.05.11	Taunus Observatory	TO 048
TO 055	2022.05.12	Taunus Observatory	TO 048
TO 058	2022.05.14	Taunus Observatory	TO 056
TO 059	2022.05.15	Taunus Observatory	TO 056
GEO 003	2022.05.03	Frankfurt Riedberg	GEO 001
GEO 007	2022.05.06	Frankfurt Riedberg	GEO 006
GEO 010	2022.05.09	Frankfurt Riedberg	GEO 006
GEO 012	2022.05.11	Frankfurt Riedberg	GEO 006
GEO 013	2022.05.12	Frankfurt Riedberg	GEO 006
GEO 016	2022.05.14	Frankfurt Riedberg	GEO 014
GEO 017	2022.05.15	Frankfurt Riedberg	GEO 014

II, Table S2, Overview Detailed Information of the Analyzed Samples

Table S2. Overview of the analyzed samples, including information about the total sampling volume, the sampling time, the filter size and the daily average PM concentration. Information about the PM were obtained from the HLNUG[1] and the Chinese government[2]. Here, the PM for TOF and Beijing TSP was estimated based on data from another time period, since no data was available.

Sample Name	Sampling Volume	Sampling Time	Filter size	PM [µg m^{-3}]	# Punches 20 µg mL^{-1}
Beij 25.1	27.3 m^{-3}	11h49m	Ø47 mm	14.7	2
Beij 25.2	27.46 m^{-3}	11h53m	Ø47 mm	13.2	2
Beij 25.3	27.2 m^{-3}	11h43m	Ø47 mm	6.4	2
Beij 25.4	27.49 m^{-3}	11h54m	Ø47 mm	18.2	2
Beij 25.5	27.92 m^{-3}	12h	Ø47 mm	112.5	1
Beij 25.6	27.37 m^{-3}	11h52m	Ø47 mm	158.5	1
Beij 25.7	27.52 m^{-3}	11h55m	Ø47 mm	57	2
Beij T.1	567 m^{-3}	11h51m	203 x 254 mm	43.8	5
Beij T.2	570 m^{-3}	11h56m	204 x 254 mm	42.6	4
Beij T.3	563 m^{-3}	11h49m	205 x 254 mm	25.7	10
Beij T.4	571 m^{-3}	11h57m	206 x 254 mm	43.6	5
Beij T.5	576 m^{-3}	12h08m	207 x 254 mm	187.9	1
Beij T.6	569 m^{-3}	11h53m	208 x 254 mm	254	1
Beij T.7	572 m^{-3}	11h58m	209 x 254 mm	133.5	4
HOC 009	720 m^{-3}	24 h	Ø150 mm	22	1
HOC 012	720 m^{-3}	24 h	Ø150 mm	12.5	2
HOC 014	720 m^{-3}	24 h	Ø150 mm	15.5	2
HOC 015	720 m^{-3}	24 h	Ø150 mm	26.6	2
HOC 016	720 m^{-3}	24 h	Ø150 mm	14.4	2
HOC 018	720 m^{-3}	24 h	Ø150 mm	15.5	2
HOC 019	720 m^{-3}	24 h	Ø150 mm	18	2
TOF 1	720 m^{-3}	12 h + 12 h	Ø150 mm	10.4	2
TOF 2	720 m^{-3}	12 h + 12 h	Ø150 mm	5	4

TOF 3	720 m^{-3}	12 h + 12 h	Ø150 mm	9.2	2
TOF 4	720 m^{-3}	12 h + 12 h	Ø150 mm	8.6	2
TOF 5	720 m^{-3}	12 h + 12 h	Ø150 mm	6.5	2
TOF 6	720 m^{-3}	12 h + 12 h	Ø150 mm	7.3	2
TOF 7	720 m^{-3}	12 h + 12 h	Ø150 mm	9.5	3
TO 045	720 m^{-3}	24 h	Ø150 mm	15.3	2
TO 049	720 m^{-3}	24 h	Ø150 mm	7.4	4
TO 052	720 m^{-3}	24 h	Ø150 mm	13.5	2
TO 054	720 m^{-3}	24 h	Ø150 mm	12.6	2
TO 055	720 m^{-3}	24 h	Ø150 mm	9.6	3
TO 058	720 m^{-3}	24 h	Ø150 mm	10.8	3
TO 059	720 m^{-3}	24 h	Ø150 mm	13.9	2
GEO 003	720 m^{-3}	24 h	Ø150 mm	21.9	1
GEO 007	720 m^{-3}	24 h	Ø150 mm	12.5	2
GEO 010	720 m^{-3}	24 h	Ø150 mm	12.5	2
GEO 012	720 m^{-3}	24 h	Ø150 mm	27.9	2
GEO 013	720 m^{-3}	24 h	Ø150 mm	13.9	2
GEO 016	720 m^{-3}	24 h	Ø150 mm	13.7	2
GEO 017	720 m^{-3}	24 h	Ø150 mm	15.2	2

III, Figure S1, Extraction Method

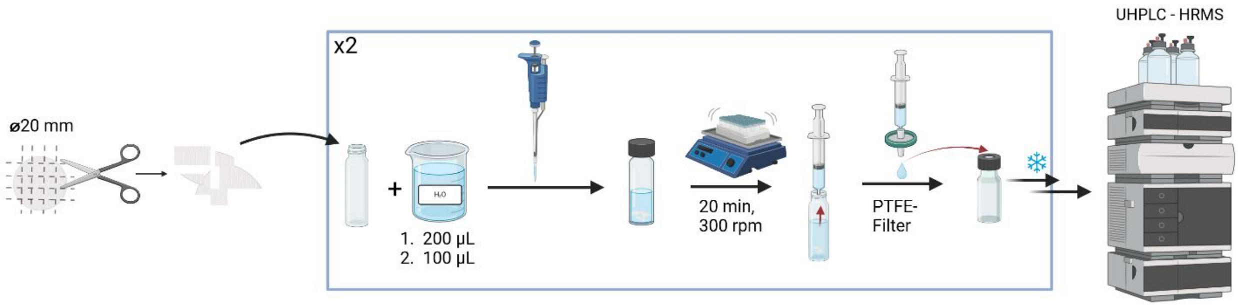

Figure S1. Schematic representation of the experimental procedure (displayed for case (a)).

Created in BioRender. Vogel, A. (2026) https://BioRender.com/hbbov6m

IV, Figure S2, UHPLC-HRMS Setup

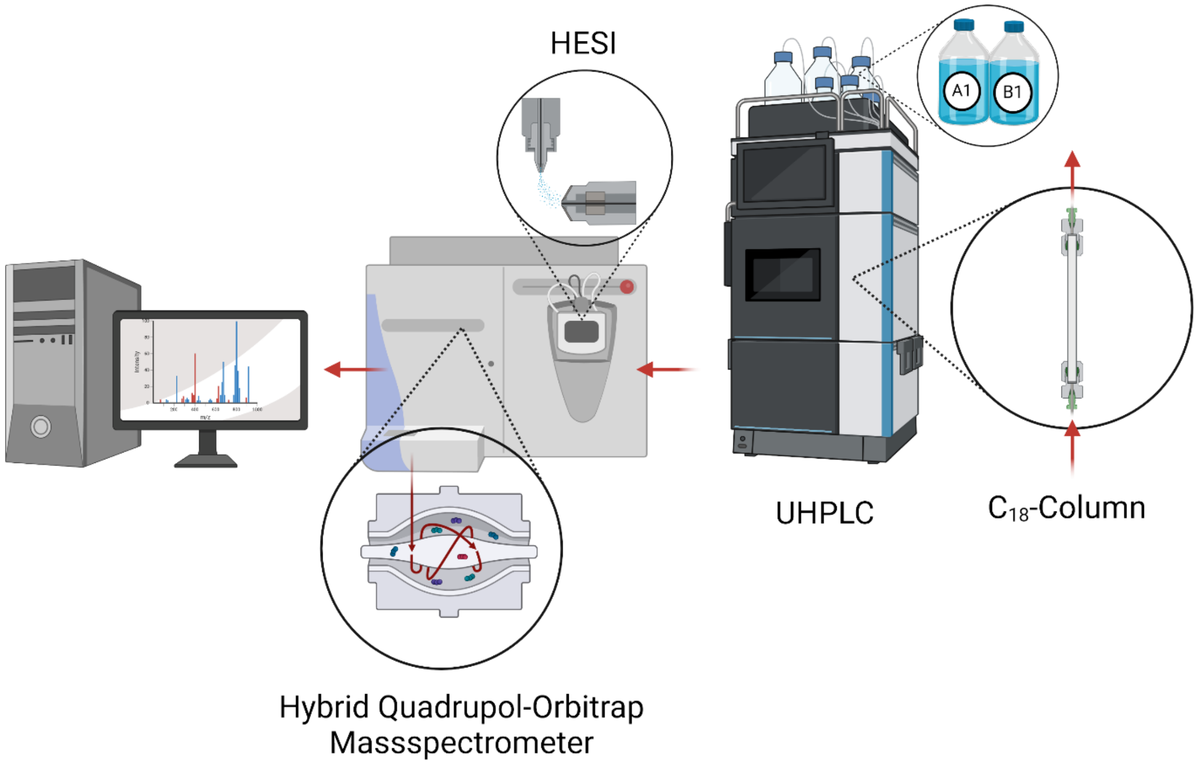

Figure S2. Schematic representation of the measurement setup, consisting of the UHPLC with the A1 and B1 mobile phase solvent, as well as a C_{18}-column, the HESI, the HRMS, and the controlling computer unit. Created in BioRender. Breuninger, A. (2026) https://BioRender.com/oa6dsm3

V, Table S3, UHPLC-HRMS Method

Table S3. Overview of the set parameters of the UHPLC-HRMS method used.

Instrument Method	
**Sampler Modus**	
Puncture Offset	2 µm
Wash Speed	20.0 [µL/s]
Injection Wash Mode	Both
Wash Time	5.0 [s]
Dispense Speed	5.000 [µL/s]
Draw Speed	5.000 [µL/s]
Temperature Control	On
Temperature Nominal	15.0 [°C]
**Post Column**	
Delta Temperature	1.00 [°C]
Temperature Control	On
Temperature Nominal	40.0 [°C]
Equilibration Time	1.0 [min]
**Column**	
Mode	
Delta Temperature	0.50 [°C]
Temperature Control	On
Temperature Nominal	40.00 [°C]
Equilibration Time	1.0 [min]
**Pump**	
Pressure lower limit	0 [bar]

Pressure upper limit	737 [bar]
Maximum Flow Ramp Up	6.00 [mL/min^2]
Maximum Flow Ramp Down	6.00 [mL/min^2]
Method	
Pump Flow Nominal	0.400 [mL/min]
0.000 min	1.0 [%] B1
0.500 min	1.0 [%] B1
14.000 min	99.0 [%] B1
16.000 min	99.0 [%] B1
17.000 min	1.0 [%] B1
20.000 min	Stop Run

Orbitrap

**Overall method settings**	
Method duration	20.00 min
Chrom. Peak width	6 s
**Experiment**	
Full MS	
Polarity	negative and positive
dd-MS2	Discovery
Resolution	70,000
#Scan ranges	1
Scan Range	70 to 750 *m/z*
AGC Target	1e6
Maximum IT	auto
Microscans	1

Spectrum data type	Profile
dd-MS2 Discovery	
Resolution	17,500
Isolation window	3.0 m/z
(N)CE / Stepped (N)CE	
Default charge state	ce: 15,30,45
AGC Target	1e5
Maximum IT	auto
Loop count	1
Minimum AGC Target	5e4
Intensity Threshold	auto
Apex trigger	0.5 to 3 s
Dynamic exclusion	auto
Exclude isotopes	on
Spectrum data type	Profile
Setup	
Lock Masses	
112.98562 (Formic Acid dimer w Na)	negative
100.07569 (N-Methyl-2pyrrolidone, floor stripper)	positive

VI, Figure S3, Quality Control of Measurement Stability

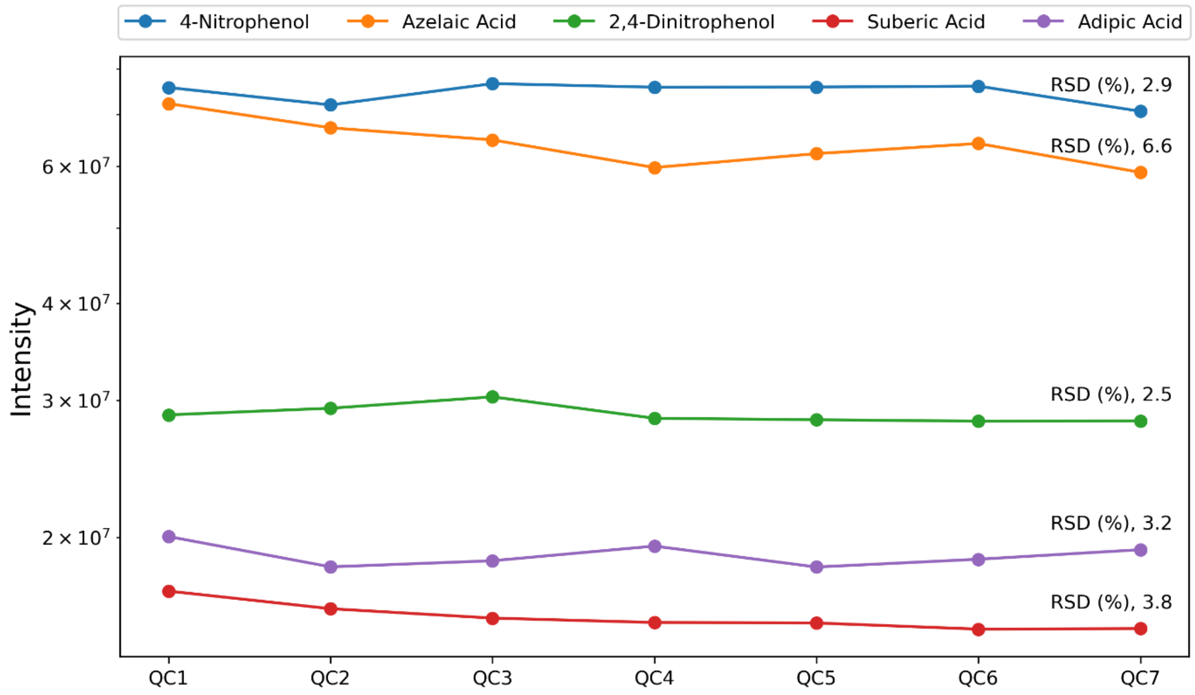

Figure S3. Variation in peak intensity of 4-nitrophenol, azelaic acid, 2,4-dinitrophenol, suberic acid and adipic acid, by measuring the same sample several times throughout the measurement series. Relative standard deviation (RSD) displayed in %.

VII, Figure S4, Mass Accuracy

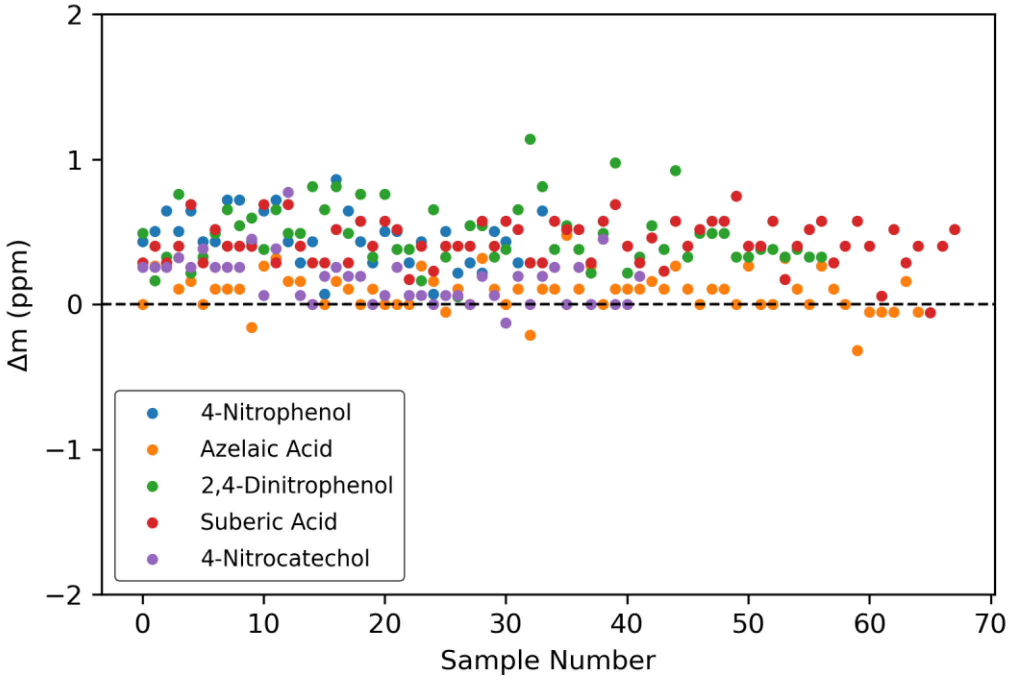

Figure S4. Mass deviation in Δm (ppm) of 4-nitrophenol, azelaic acid, 2,4-dinitrophenol, suberic acid and 4-nitrocatechol.

VIII, Figure S5, OP Measurements

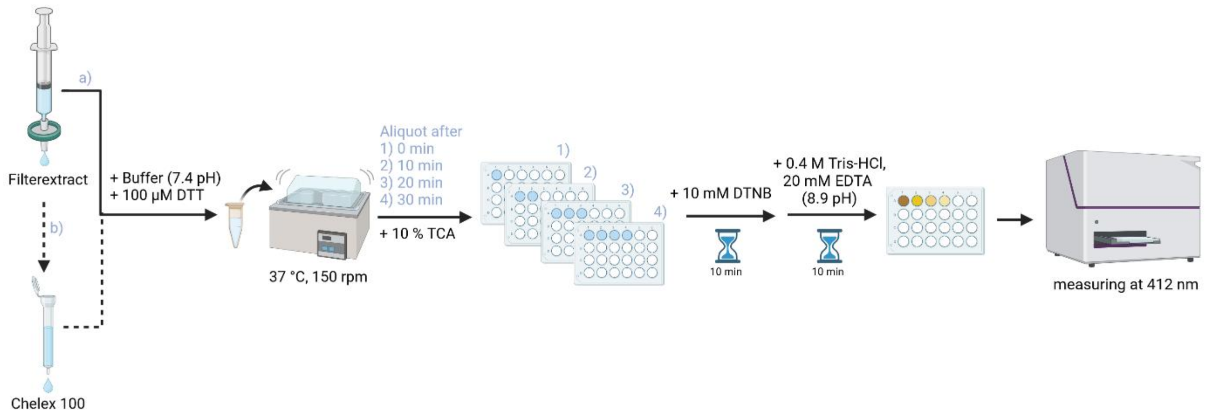

Figure S5. Schematic representation of the experimental procedure used for measuring the oxidative potential. a) Procedure without Chelex-treatment, b) Procedure with Chelex-treatment. Created in BioRender. Vogel, A. (2026) https://BioRender.com/mv4a1gi

IX, OP^{DTT}-Protocol, detailed

Chemicals used. Acetonitrile (ACN, HPLC grade), ethylenediamine tetraacetic acid (EDTA, 99%), tris-base (molecular biology grade, 99,5%), Hydrochloric acid (HCl, Honeywell Fluka™) from *Fisher Scientific*. Ultrapure water (MilliQ Reference A+), dipotassiumhydratephosphate (K_2HPO_4, 99%), dihydratepotassiumphosphate (KH_2PO_4, 99.5%), 9,10-phenanthrenquinone (PQN, 99%), trichloricacetic acid (TCA, 99%), copper(II)sulfate pentahydrate ($CuSO_4 \cdot 5H_2O$, dithiothreitol (DTT, MQ100), 5,5-dithiobis-2-nitrobenzoic acid (DTNB, 98%), Chelex® 100 resin from *Merck KGaA*.

DTT-Assay. For measuring the oxidative potential, an in-vial PM concentration of ~20 µg mL^{-1} was used. The number of punches (14.2 mm diameter) taken from each filter that results in the required concentration can be found in Table S2. The extraction was carried out as described in Material and Methods. A total of 10 mL was extracted. 6 mL of the extract were treated with Chelex® 100 resin (200-400 mesh particle size, *Merck*), by passing the extract through the column (see Figure 4 b)). The column was prepared by packing a polypropylene gravity flow column (6 mL with 20 µM frits, *Marvelgent*) with ~0.8–0.9 g Chelex® 100 resin and pre-rinsing with 500 mL MilliQ water to decrease the effluent pH to 7–8. The following steps were applied for the Chelex-treated extracts as for the untreated extract.

All steps were carried out under light avoidance and performed in replicates, transferring 1.2 mL extract each to an amber coloured 1.5 mL vial. 0.15 mL 1 M potassium phosphate buffer and 0.15 mL 1000 µM DTT were added. The vials were placed in an incubator (12 L Shaking Bath, *VWR*) at 37 °C and 150 rpm. After either 0, 10, 20 and 30 min or 0, 15, 30 and 45 min, an aliquot of 0.25 mL was taken out and added to 0.25 mL 10% TCA on a 24-well microplate. After every time point has been quenched, 25 µL 10 mM DTNB was added to each sample. After 10 min, 1 mL 0.4 M Tris-

Base (pH adjusted to 8.9 with HCl) with 20 mM EDTA was added. After another 10 min, the samples were ready for measurement.

Positive Control. To check the methods' performance, a positive control was performed every week. Therefore, 25 µL 12.5 µM 9,10-phenanthrenquinone (99%, *Merck KGaA*) (PQN) were transferred to an amber-coloured vial and 2.7 mL 0.1 M potassium phosphate buffer and 0.3 mL 1000 µM DTT were added. The vial was placed in an incubator at 37 °C and 150 rpm.

Reagent Background. For each measurement the absorbance, which occurs through the chemicals and solutions used, was measured. For this purpose, DTT was replaced by MilliQ while everything else was kept as in the procedures described. As no DTT is available, the chemicals were directly placed on the 24-well microplate and no incubation was carried out.

Calibration Curve. To convert the measured OD to a DTT concentration, a calibration curve was made. For that, various DTT standard solutions (10, 25, 50, 75, 100 µM) are made in 0.1 M potassium phosphate buffer. An aliquot of 0.25 mL of each standard solution was added to 0.25 mL TCA and 25 µL DTNB were added. After 10 min, 1 mL 0.4 M Tris-Base with 20 mM EDTA was added. After another 10 min, the samples were ready for measurement.
As the DTT stock solution at a concentration of 1000 µM was prepared anew on a weekly basis, a fresh calibration was also conducted weekly.

X, Figure S6, Positive Control

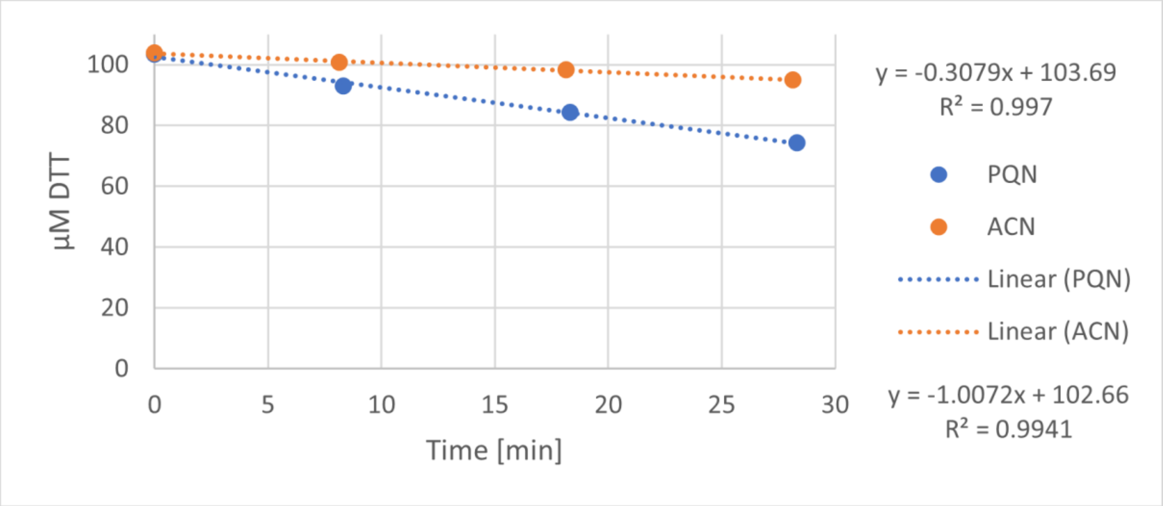

Figure S6. Representation of the positive control, which was conducted on a weekly basis. Blue, the solution with PQN, orange, the solvent blank. The slope represents the DTT consumption in µM DTT min^{-1}.

XI, Compound Discoverer Workflow, negative mode

Search name: 20230627*_LC_Measurements_neg_all*

Search description: -

Search date: 6/29/2023 9:45:01 AM

Created with Discoverer version: 3.3.2.31

Input Files (0)

–>Select Spectra (2)

Select Spectra (2)

–>Align Retention Times (ChromAlign) (23)

Align Retention Times (ChromAlign) (23)

–>Detect Compounds (21)

Detect Compounds (21)

–>Group Compounds (4)

Group Compounds (4)

–>Fill Gaps (17)

–>Assign Compound Annotations (11)

–>Compound Class Scoring (35)

–>Calculate Mass Defect (37)

–>Search mzCloud (31)

–>Search mzVault (32)

–>Predict Compositions (5)

Fill Gaps (17)

–>Mark Background Compounds (18)

Mark Background Compounds (18)

Assign Compound Annotations (11)

Compound Class Scoring (35)

Calculate Mass Defect (37)

Search mzCloud (31)

Search mzVault (32)

Predict Compositions (5)

Descriptive Statistics (24) Differential Analysis (25)

Processing node 2: Select Spectra

---

1. Spectrum Properties Filter:

Lower RT Limit: 1.2

Upper RT Limit: 17.5

First Scan: 0

Last Scan: 0

Ignore Specified Scans: (not specified)

Lowest Charge State: 0

Highest Charge State: 0

Min. Precursor Mass: 50 Da

Max. Precursor Mass: 5000 Da

Total Intensity Threshold: 1000

Minimum Peak Count: 1

2. Scan Event Filters:

Mass Analyzer: Is FTMS

MS Order: Any

Activation Type: Is HCD

Min. Collision Energy: 0

Max. Collision Energy: 1000

Scan Type: Any

Polarity Mode: Is -

MS1 Mass Range: (not specified)

FAIMS CV: (not specified)

3. Peak Filters:

- S/N Threshold (FT-only): 5

4. Replacements for Unrecognized Properties:

Unrecognized Charge Replacements: 1

Unrecognized Mass Analyzer Replacements: ITMS

Unrecognized MS Order Replacements: MS2

Unrecognized Activation Type Replacements: HCD

Unrecognized Polarity Replacements: - Unrecognized MS Resolution@200 Replacements: 60000 - Unrecognized MSn Resolution@200 Replacements: 30000

5. General Settings:

Precursor Selection: Use MS1 Precursor

Use Isotope Pattern in Precursor Reevaluation: True

Provide Profile Spectra: Automatic

Store Chromatograms: False

Processing node 23: Align Retention Times (ChromAlign)

---

1. General Settings:

- Reference File: 220606_QC_1_neg01

Processing node 21: Detect Compounds

---

1. General Settings:

Mass Tolerance (ppm): 5 ppm

Min. Peak Intensity: 10000

Min. # Scans per Peak: 5

Use Most Intense Isotope Only: True

2. Trace Detection:

- Max. Number of Gaps to Correct: 2 - Min. Number of Adjacent Non-Zeros: 2

3. Peak Detection:

Chromatographic S/N Threshold: 1.5

Remove Baseline: False

Gap Ratio Threshold: 0.35

Max. Peak Width (min): 0.2

Min. Relative Valley Depth: 0.2

4. Isotope Pattern Detection:

Group Isotopes for: Br; Cl

Use Peak Quality for Isotope Grouping: True

Filter out Features with Bad Peaks Only: True

Zig-Zag Index Threshold: 0.2

Jaggedness Threshold: 0.4

Modality Threshold: 0.9

Remove Potentially False Positive Isotopes: True

5. Compound Detection:

Ions: [2M-H]-1; [M-CO2-H]-1; [M-H]-1; [M-H-H2O]-1

Base Ions: [M-H]-1

Remove Singlets: True

6. AcquireX Settings:

- Detect Persistent Background Ions: False

- Area Contribution: 3

- CV Contribution: 0

- FWHM to Base Contribution: 5

- Jaggedness Contribution: 5

- Modality Contribution: 5

- Zig-Zag Index Contribution: 5

Processing node 4: Group Compounds

---

1. General Settings:

Mass Tolerance: 2 ppm

RT Tolerance [min]: 0.2

Align Peaks: False

Preferred Ions: [M-H]-1

Area Integration: Most Common Ion

Peak Rating Contributions:

Peak Rating Filter:

Peak Rating Threshold: 5

Number of Files: 2

Processing node 17: Fill Gaps

1. General Settings:

Mass Tolerance: 2 ppm

S/N Threshold: 5

Use Real Peak Detection: True

Apply Restrictive Gap Filling: True

Processing node 18: Mark Background Compounds

1. General Settings:

Max. Sample/Blank: 5

Max. Blank/Sample: 0

Hide Background: True

Processing node 11: Assign Compound Annotations

1. General Settings:

- Mass Tolerance: 2 ppm

2. Data Sources: - Data Source #1: Predicted Compositions

Data Source #2: mzVault Search

Data Source #3: mzCloud Search (Compound Class)

Data Source #4: (not specified)

Data Source #5: (not specified)

Data Source #6: (not specified)

Data Source #7: (not specified)

3. Scoring Rules:

Use mzLogic: True

Use Spectral Distance: True

SFit Threshold: 20

SFit Range: 20

4. Reprocessing:

- Clear Names: True

Processing node 35: Compound Class Scoring

---

1. General Settings:

Compound Classes: R-OSO3, R-SO3.cLib|R-NO3.cLib

S/N Threshold: 50

High Acc. Mass Tolerance: 15 ppm

Low Acc. Mass Tolerance: 0.5 Da

Use Full MS Tree: True

Allow DIA Scoring: True

Processing node 37: Calculate Mass Defect

---

1. Mass Defect:

Fractional Mass: False

Standard Mass Defect: True

Relative Mass Defect: False

Kendrick Mass Defect: True - Nominal Mass Rounding: Round

2. Kendrick Formula:

Formula 1: C H2

Formula 2: (not specified)

Formula 3: (not specified)

Formula 4: (not specified)

Formula 5: (not specified)

Processing node 31: Search mzCloud

---

1. General Settings:

Compound Classes: All

Precursor Mass Tolerance: 10 ppm

FT Fragment Mass Tolerance: 10 ppm

IT Fragment Mass Tolerance: 0.4 Da

Library: Autoprocessed; Reference

Post Processing: Recalibrated

Max. # Results: 10

Annotate Matching Fragments: True

Search MSn Tree: False

2. DDA Search:

Identity Search: HighChem HighRes

Match Activation Type: True

Match Activation Energy: Match with Tolerance

Activation Energy Tolerance: 20

Apply Intensity Threshold: True

Similarity Search: None

Match Factor Threshold: 60

3. DIA Search:

Use DIA Scans for Search: False

Max. Isolation Width [Da]: 500

Match Activation Type: False

Match Activation Energy: Any

Activation Energy Tolerance: 100 - Apply Intensity Threshold: False

Match Factor Threshold: 20

Processing node 32: Search mzVault

---

1. Search Settings:

mzVault Library: AP_O3_rA_MW_neg.db| AP_O3_UV_rA_MW_neg.db|

BCY_O3_rA_MW_neg.db|

BCY_O3_UV_rA_MW_neg.db| BP_O3_rA_MW_neg.db| BP_O3_UV_rA_MW_neg.db|

D3C_O3_rA_MW_neg.db| D3C_O3_UV_rA_MW_neg.db| LIM_O3_rA_MW_neg.db|

LIM_O3_UV_rA_MW_neg.db| NAP_O3_UV_rA_MW_neg.db|

TMB_O3_UV_rA_MW_neg.db|

TOL_O3_UV_rA_MW_neg.db| XYL_O3_UV_rA_MW_neg.db

Max. # Results: 10

Match Factor Threshold: 80

Search Algorithm: HighChem HighRes

Match Analyzer Type: True

IT Fragment Mass Tolerance: 0.4 Da

FT Fragment Mass Tolerance: 10 ppm

Use Retention Time: True

Precursor Mass Tolerance: 10 ppm

Apply Intensity Threshold: True

Match Ionization Method: True

Ion Activation Energy Tolerance: 20

Match Ion Activation Energy: Match with Tolerance

Match Ion Activation Type: True

Compound Classes: All

Remove Precursor Ion: True

RT Tolerance [min]: 1

Processing node 5: Predict Compositions

---

1. Prediction Settings:

Mass Tolerance: 2 ppm

Min. Element Counts: C H

Max. Element Counts: C90 H190 Br4 Cl4 N6 O20 P S6

Min. RDBE: 0

Max. RDBE: 40

Min. H/C: 0.1

Max. H/C: 3.5

Max. # Candidates: 10

Max. # Internal Candidates: 200

2. Pattern Matching:

Intensity Tolerance [%]: 30

Intensity Threshold [%]: 0.1

S/N Threshold: 5

Min. Spectral Fit [%]: 30

Min. Pattern Cov. [%]: 90

Use Dynamic Recalibration: True

3. Fragments Matching:

Use Fragments Matching: True

Mass Tolerance: 2 ppm

S/N Threshold: 5

Processing node 24: Descriptive Statistics

No parameters

Processing node 25: Differential Analysis

General Settings: - Log10 Transform Values: True

Peak Rating Contributions:

- Update Peak Rating: True

- Area Contribution: 3

- CV Contribution: 0

- FWHM to Base Contribution: 5

- Jaggedness Contribution: 5

- Modality Contribution: 5

- Zig-Zag Index Contribution: 5

XII, Compound Discoverer Workflow, positive mode

Search name: 20232906_*HPLC_all_pos*

Search description: -

Search date: 6/29/2023 9:57:30 AM

Created with Discoverer version: 3.3.2.31

Input Files (0)

–>Select Spectra (2)

Select Spectra (2)

–>Align Retention Times (ChromAlign) (23)

Align Retention Times (ChromAlign) (23)

–>Detect Compounds (21)

Detect Compounds (21)

–>Group Compounds (4)

Group Compounds (4)

–>Fill Gaps (17)

–>Assign Compound Annotations (11)

–>Compound Class Scoring (35)

–>Calculate Mass Defect (37)

–>Search mzCloud (31)

–>Search mzVault (32)

–>Predict Compositions (5)

Fill Gaps (17)

–>Mark Background Compounds (18)

Mark Background Compounds (18)

Assign Compound Annotations (11)

Compound Class Scoring (35)

Calculate Mass Defect (37)

Search mzCloud (31)

Search mzVault (32)

Predict Compositions (5)

Descriptive Statistics (24) Differential Analysis (25)

Processing node 2: Select Spectra

---

1. Spectrum Properties Filter:

- Lower RT Limit: 1.2

- Upper RT Limit: 17.5

- First Scan: 0

- Last Scan: 0

- Ignore Specified Scans: (not specified)

- Lowest Charge State: 0

- Highest Charge State: 0

- Min. Precursor Mass: 50 Da

- Max. Precursor Mass: 5000 Da

- Total Intensity Threshold: 1000

- Minimum Peak Count: 1

2. Scan Event Filters:

- Mass Analyzer: Is FTMS

- MS Order: Any

- Activation Type: Is HCD

- Min. Collision Energy: 0

- Max. Collision Energy: 1000

- Scan Type: Any

- Polarity Mode: Is +

- MS1 Mass Range: (not specified)

- FAIMS CV: (not specified)

3. Peak Filters:

- S/N Threshold (FT-only): 5

4. Replacements for Unrecognized Properties:

- Unrecognized Charge Replacements: 1

- Unrecognized Mass Analyzer Replacements: ITMS

- Unrecognized MS Order Replacements: MS2

- Unrecognized Activation Type Replacements: HCD

- Unrecognized Polarity Replacements: - Unrecognized MS Resolution@200 Replacements: 60000 - Unrecognized MSn Resolution@200 Replacements: 30000

5. General Settings:

- Precursor Selection: Use MS1 Precursor

- Use Isotope Pattern in Precursor Reevaluation: True

- Provide Profile Spectra: Automatic

- Store Chromatograms: False

Processing node 23: Align Retention Times (ChromAlign)

---

1. General Settings:

- Reference File: 220606_QC_1_pos01

Processing node 21: Detect Compounds

---

1. General Settings:

- Mass Tolerance [ppm]: 5 ppm

- Min. Peak Intensity: 50000

- Min. # Scans per Peak: 5

- Use Most Intense Isotope Only: True

2. Trace Detection:

- Max. Number of Gaps to Correct: 2 - Min. Number of Adjacent Non-Zeros: 2

3. Peak Detection:

- Chromatographic S/N Threshold: 1.5

- Remove Baseline: False

- Gap Ratio Threshold: 0.35

- Max. Peak Width [min]: 0.2

- Min. Relative Valley Depth: 0.2

4. Isotope Pattern Detection:

- Group Isotopes for: Br; Cl

- Use Peak Quality for Isotope Grouping: True

- Filter out Features with Bad Peaks Only: True

- Zig-Zag Index Threshold: 0.2

- Jaggedness Threshold: 0.4

- Modality Threshold: 0.9

- Remove Potentially False Positive Isotopes: True

5. Compound Detection:

- Ions: [M+H]+1; [M+K]+1; [M+Na]+1; [M+NH4]+1

- Base Ions: [M+H]+1

- Remove Singlets: True

6. AcquireX Settings:

- Detect Persistent Background Ions: False

Processing node 4: Group Compounds

---

1. General Settings:

- Mass Tolerance: 2 ppm - RT Tolerance [min]: 0.2
- Align Peaks: False

- Preferred Ions: [M+H]+1

- Area Integration: Most Common Ion

2. Peak Rating Contributions:

- Area Contribution: 3

- CV Contribution: 0

- FWHM to Base Contribution: 5

- Jaggedness Contribution: 5

- Modality Contribution: 5

- Zig-Zag Index Contribution: 5

3. Peak Rating Filter:

- Peak Rating Threshold: 5

- Number of Files: 2

Processing node 17: Fill Gaps

---

1. General Settings:

- Mass Tolerance: 2 ppm

- S/N Threshold: 5

- Use Real Peak Detection: True

- Apply Restrictive Gap Filling: True

Processing node 18: Mark Background Compounds

---

1. General Settings:

- Max. Sample/Blank: 5

- Max. Blank/Sample: 0

- Hide Background: True

Processing node 11: Assign Compound Annotations

---

1. General Settings: - Mass Tolerance: 2 ppm

2. Data Sources:

- Data Source #1: Predicted Compositions

- Data Source #2: mzVault Search

- Data Source #3: mzCloud Search (Compound Class)

- Data Source #4: (not specified)

- Data Source #5: (not specified)

- Data Source #6: (not specified)

- Data Source #7: (not specified)

3. Scoring Rules:

- Use mzLogic: True

- Use Spectral Distance: True

- SFit Threshold: 20

- SFit Range: 20

4. Reprocessing:

- Clear Names: True

Processing node 35: Compound Class Scoring

---

1. General Settings:

- Compound Classes: R-OSO3, R-SO3.cLib|R-NO3.cLib

- S/N Threshold: 50

- High Acc. Mass Tolerance: 15 ppm

- Low Acc. Mass Tolerance: 0.5 Da

- Use Full MS Tree: True

- Allow DIA Scoring: True

Processing node 37: Calculate Mass Defect

---

1. Mass Defect:

- Fractional Mass: False
- Standard Mass Defect: True
- Relative Mass Defect: False
- Kendrick Mass Defect: True - Nominal Mass Rounding: Round

2. Kendrick Formula:

- Formula 1: C H2
- Formula 2: (not specified)
- Formula 3: (not specified)
- Formula 4: (not specified)
- Formula 5: (not specified)

Processing node 31: Search mzCloud

---

1. General Settings:

- Compound Classes: All
- Precursor Mass Tolerance: 10 ppm
- FT Fragment Mass Tolerance: 10 ppm
- IT Fragment Mass Tolerance: 0.4 Da
- Library: Autoprocessed; Reference
- Post Processing: Recalibrated
- Max. # Results: 10

- Annotate Matching Fragments: True

- Search MSn Tree: False

2. DDA Search:

- Identity Search: HighChem HighRes

- Match Activation Type: True

- Match Activation Energy: Match with Tolerance

- Activation Energy Tolerance: 20

- Apply Intensity Threshold: True

- Similarity Search: None - Match Factor Threshold: 60

3. DIA Search:

- Use DIA Scans for Search: False

- Max. Isolation Width [Da]: 500

- Match Activation Type: False

- Match Activation Energy: Any

- Activation Energy Tolerance: 100 - Apply Intensity Threshold: False
- Match Factor Threshold: 20

Processing node 32: Search mzVault

---

1. Search Settings:

- mzVault Library:

AP_O3_rA_MW_neg.db|AP_O3_UV_rA_MW_neg.db|BCY_O3_rA_MW_neg.db|

BCY_O3_UV_rA_MW_neg.db|BP_O3_rA_MW_neg.db|BP_O3_UV_rA_MW_neg.db|

D3C_O3_rA_MW_neg.db|D3C_O3_UV_rA_MW_neg.db|LIM_O3_rA_MW_neg.db|

LIM_O3_UV_rA_MW_neg.db|NAP_O3_UV_rA_MW_neg.db|TMB_O3_UV_rA_MW_neg.db|

TOL_O3_UV_rA_MW_neg.db|XYL_O3_UV_rA_MW_neg.db

- Max. # Results: 10

- Match Factor Threshold: 80

- Search Algorithm: HighChem HighRes

- Match Analyzer Type: True

- IT Fragment Mass Tolerance: 0.4 Da

- FT Fragment Mass Tolerance: 10 ppm

- Use Retention Time: True

- Precursor Mass Tolerance: 10 ppm

- Apply Intensity Threshold: True

- Match Ionization Method: True

- Ion Activation Energy Tolerance: 20

- Match Ion Activation Energy: Match with Tolerance

- Match Ion Activation Type: True

- Compound Classes: All

- Remove Precursor Ion: True

- RT Tolerance [min]: 1

Processing node 5: Predict Compositions

---

1. Prediction Settings:

- Mass Tolerance: 2 ppm

- Min. Element Counts: H

- Max. Element Counts: C90 H190 Br4 Cl4 N6 O20 P S6

- Min. RDBE: 0

- Max. RDBE: 40

- Min. H/C: 0.1

- Max. H/C: 3.5

- Max. # Candidates: 10

- Max. # Internal Candidates: 200

2. Pattern Matching:

- Intensity Tolerance [%]: 30

- Intensity Threshold [%]: 0.1

- S/N Threshold: 5

- Min. Spectral Fit [%]: 30

- Min. Pattern Cov. [%]: 90

- Use Dynamic Recalibration: True

3. Fragments Matching:

- Use Fragments Matching: True

- Mass Tolerance: 2 ppm

- S/N Threshold: 5

Processing node 24: Descriptive Statistics

No parameters

Processing node 25: Differential Analysis

---

1. General Settings: - Log10 Transform Values: True

2. Peak Rating Contributions:

- Update Peak Rating: True
- Area Contribution: 3
- CV Contribution: 0
- FWHM to Base Contribution: 5
- Jaggedness Contribution: 5
- Modality Contribution: 5
- Zig-Zag Index Contribution: 5

XIII, Aromaticity Equivalent according to Yassine et al.[3]

$$DBE = \#C - \frac{\#H}{2} + \frac{\#N}{2} + 1 \quad \textbf{(1)}$$

$$Xc = \frac{3*[DBE-(m*\#O+n*\#S)]-2}{DBE-(m*\#O+n*\#S)} \textit{ (0 if < 0)} \quad \textbf{(2)}$$

With:

DBE:	Double bond equivalent
#H:	Number of hydrogen atoms
#C:	Number of carbon atoms
#O:	Number of oxygen atoms
#N:	Number of nitrogen atoms
#S:	Number of sulphur atoms
m and *n*:	Fractions of O and S atoms in π-bonds, presumed to be 0.5[3]
Xc:	Aromaticity equivalent<br>$Xc < 2.5000$: nonaromatic compounds<br>$2.5000 \leq Xc \leq 2.7143$: aromatic compounds<br>$Xc \geq 2.7143$: condensed aromatics

XIV, z-Transformation and Euclidean Distance

$$d_{k,l} = \sqrt[2]{\sum_{j=1}^{J} \left(x_{k,j} - x_{l,j}\right)^2} \quad \textbf{(3)}$$

$$z_{if} = \frac{x_{if} - m_f}{s_f} \quad \textbf{(4)}$$

With:

z_{if}:	Standardized data of matrix type i x f
x_{if}:	Object in matrix
m_f:	Mean value
s_f:	Mean absolute deviation
$d_{k,l}$:	Distance between objects k and l
$x_{k,j}$, $x_{l,j}$:	Value for variable j for object k and l

XV, Figure S7, DTT Calibration Curve

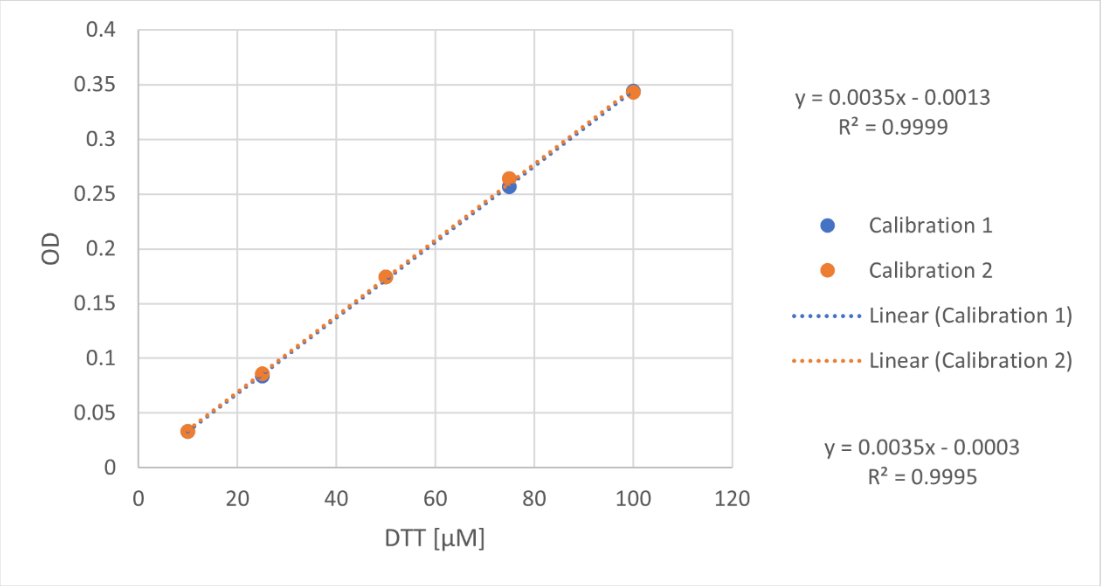

Figure S7. Representation of the positive control, which was conducted on a weekly basis. Blue, the solution with PQN, orange, the solvent blank. The slope represents the DTT consumption in µM DTT min^{-1}.

$$y = m_{calib} \cdot x + b_{calib} \quad \textbf{(7)}$$

$$x = \frac{y - b_{calib}}{m_{calib}} \quad \textbf{(8)}$$

With:

y:	Measured absorbance in OD
b_{calib}:	x-axis intercept of calibration curve
m_{calib}:	Slope of calibration curve
x:	DTT concentration in µM

XVI, Calculation of the Metal Concentration

$$\frac{\left(\frac{I(i)}{I(In)}\right)_{Std}}{\left(\frac{c(i)}{c(In)}\right)_{Std}} = \frac{\left(\frac{I(i)}{I(In)}\right)_{Samp}}{\left(\frac{c(i)}{c(In)}\right)_{Samp}} \qquad \textbf{(5)}$$

$$\frac{\left(\frac{I(i)}{I(In)}\right)_{Samp} \cdot c(In)_{samp} \cdot c(i)_{std}}{\left(\frac{I(i)}{I(In)}\right)_{Samp} \cdot c(In)_{Std}} = c(i)_{Samp} \qquad \textbf{(6)}$$

With:

I(i):	Intensity of element *i*
c(i):	Concentration of element *i* in ppb
i_{Std}:	Element in standard solution
i_{Samp}:	Element in sample
$c(In)_{samp}$:	Concentration of indium in the sample = 5 ppb
$c(In)_{Std}$:	Concentration of indium in the standard solution = 10.48 ppb

XVII, Table S4, Standard Solution for ICP-MS

Table S4. Composition of the standard solution used for ICP-MS measurements.

Fe:	5.25 ppb
Mn:	5.25 ppb
Pb:	10.48 ppb
In:	10.48 ppb
Zn:	5.25 ppb
Cu:	5.25 ppb

XVIII, Normalization according to Air Volume

The values used for the calculation can be found in Table S1 and Table S2. The result corresponds to a concentration in ng m^{-3}.

$$N_x = \frac{A_{x,punch}}{A_{x,filter}} \cdot V_{x,Air,total} \tag{9}$$

$$c(i)_{Samp,norm} = \frac{m(i)_{Samp}}{N_x} \tag{10}$$

With:

N_x:	Normalization factor of sample *x* in m
$A_{x,punch}$:	Taken punch area of sample *x* in mm^2
$A_{x,filter}$:	Area of the filter sample *x* in mm^2
$V_{x,Air,total}$:	Sampled air volume of sample *x* in m^3
$m(i)_{Samp}$:	Mass of element *i* in the sample in ng

XIX, OP Mass and Volume Normalization

$$OP_M = \frac{OP}{PM_{vial}} \cdot 1000 \quad \textbf{(11)}$$

$$PM_{Vial} = \frac{PM_{filter,total} \cdot \frac{A_{punch} \cdot \#_{punch}}{A_{x,filter}}}{V_{extract}} \cdot V_{Sample} \quad \textbf{(12)}$$

$$OP_V = \frac{OP}{V_{Air,Vial}} \quad \textbf{(13)}$$

$$V_{Air,Vial} = \frac{V_{x,Air,total} \cdot \frac{A_{punch} \cdot \#_{punch}}{A_{x,filter}}}{V_{extr}} \quad \textbf{(14)}$$

With:

OP_M:	Oxidative potential normalized by mass in pmol DTT $min^{-1}\mu g^{-1}$
PM_{vial}:	PM in the incubation vial in µg
$PM_{filter,total}$:	Total PM collected on the filter in µg
A_{punch}:	Punch area = 158.37 mm^2
$A_{x,filter}$:	Area of the filter sample x in mm^2
$\#_{punch}$:	Number of punches taken
$V_{extract}$:	Extracted volume = 10 mL
V_{Sample}:	Sample volume used for measurement = 1.2 mL
OP_V:	Oxidative potential normalized by air volume in nmol DTT $min^{-1}m^{-3}$
$V_{x,Air,total}$:	Sampled air volume of sample x m^{-3}

XX, Table S5, Metal Concentrations and theoretical $OP_{V,metal}$

Table S5. Metal concentrations [ng m^{-3}] and, the measured WS OP_V, the WS OP_V of chelexed samples ($OP_{V, Cx}$) and theoretical $OP_{V,metal}$ [all in nmol DTT $min^{-1}m^{-3}$] which derived from calculations according to metal OP^{DTT}, measured by Charrier and Antassio.[5]

	Metal concentration								
Sample	Mn	Fe	Cu	Zn	Pb	OP_V	$OP_{V, Cx}$	$OP_{V, metal}$	contribution to WS OP_V [%]
Beij 25.1	0.57	2.49	1.30	0.98	0.08	1.35	0.59	0.003	0.21
Beij 25.2	0.09	0.50	0.57	0.19	0.06	0.36	0.45	0.001	0.28
Beij 25.3	0.13	0.00	0.15	0.00	0.00	NaN	NaN	0.000	0.00
Beij 25.4	0.24	1.74	0.27	1.32	0.00	0.89	0.67	0.001	0.10
Beij 25.5	6.76	14.36	3.84	36.93	2.23	2.02	2.46	0.015	0.74
Beij 25.6	8.46	18.13	4.28	45.98	2.40	2.16	1.64	0.018	0.83
Beij 25.7	1.19	4.06	2.10	3.21	0.52	0.83	0.89	0.005	0.59
Beij T.1	0.81	1.74	16.00	0.00	0.07	0.98	0.58	0.026	2.65
Beij T.2	0.65	0.04	20.87	0.00	0.07	1.15	0.79	0.032	2.81
Beij T.3	0.11	1.23	9.99	0.00	0.00	0.41	0.37	0.018	4.31
Beij T.4	0.28	0.77	25.42	0.04	0.05	0.92	0.60	0.040	4.31
Beij T.5	22.00	8.04	425.15	8.33	0.54	4.69	4.00	0.616	13.13
Beij T.6	32.80	11.52	430.21	21.98	1.58	4.53	4.17	0.636	14.04
Beij T.7	11.69	0.66	199.38	0.29	0.02	1.68	1.76	0.314	22.08
GEO 003	2.26	2.06	1.68	1.81	0.13	1.14	0.76	0.005	0.42
GEO 007	0.17	0.28	0.91	1.20	0.01	0.69	0.26	0.002	0.54
GEO 010	0.68	0.89	1.04	0.40	0.07	0.65	0.47	0.003	0.79
GEO 012	1.25	0.95	1.06	0.51	0.15	0.83	0.40	0.003	1.05

GEO 013	0.10	0.41	0.31	0.00	0.02	0.58	0.32	0.001	0.20
GEO 016	0.33	0.42	1.05	0.36	0.02	0.48	0.59	0.002	0.38
GEO 017	1.03	1.41	0.97	1.01	0.11	0.51	0.42	0.003	0.58
HÖC 009	1.77	2.38	1.87	6.84	0.14	0.80	0.68	0.005	0.44
HÖC 012	0.26	0.27	0.81	0.11	0.00	0.29	0.26	0.002	0.24
HÖC 014	0.79	0.63	1.24	0.17	0.07	0.19	0.16	0.003	0.45
HÖC 015	1.31	0.53	1.28	0.98	0.00	0.46	0.46	0.004	0.43
HÖC 016	0.37	0.15	0.47	0.00	0.00	0.13	0.20	0.001	0.21
HÖC 018	0.47	0.14	0.80	0.00	0.02	0.33	0.41	0.002	0.38
HÖC 019	1.10	0.49	0.93	0.24	0.09	0.37	0.28	0.003	0.55
TO 045	1.67	2.95	0.82	6.73	0.37	0.33	0.53	0.004	1.09
TO 049	0.21	0.22	0.30	0.03	0.00	0.17	0.09	0.001	0.47
TO 052	0.74	1.27	0.65	1.18	0.12	0.49	0.41	0.002	0.41
TO 054	0.73	0.58	0.37	0.28	0.22	0.20	0.12	0.002	0.73
TO 055	0.08	0.37	0.22	0.08	0.02	0.32	0.27	0.001	0.16
TO 058	0.33	0.41	0.34	0.01	0.01	0.23	0.31	0.001	0.44
TO 059	0.65	0.34	0.41	0.10	0.03	0.28	0.26	0.001	0.51
TOF 1	0.35	1.47	0.41	0.46	0.17	1.26	0.75	0.001	0.15
TOF 2	0.04	7.67	0.42	4.18	0.58	0.31	0.33	0.002	0.60
TOF 3	0.05	0.33	0.24	0.06	0.19	0.32	0.70	0.001	0.28
TOF 4	0.09	0.24	0.28	0.00	0.06	0.31	0.34	0.001	0.13
TOF 5	0.03	0.18	0.13	0.00	0.01	0.33	0.52	0.000	0.20
TOF 6	0.03	1.88	0.10	0.00	0.01	0.55	0.29	0.000	0.12
TOF 7	0.06	0.59	0.24	0.00	0.02	0.50	0.43	0.001	0.15

XXI, Figure S8, Metal-Removal by Chelex Resin

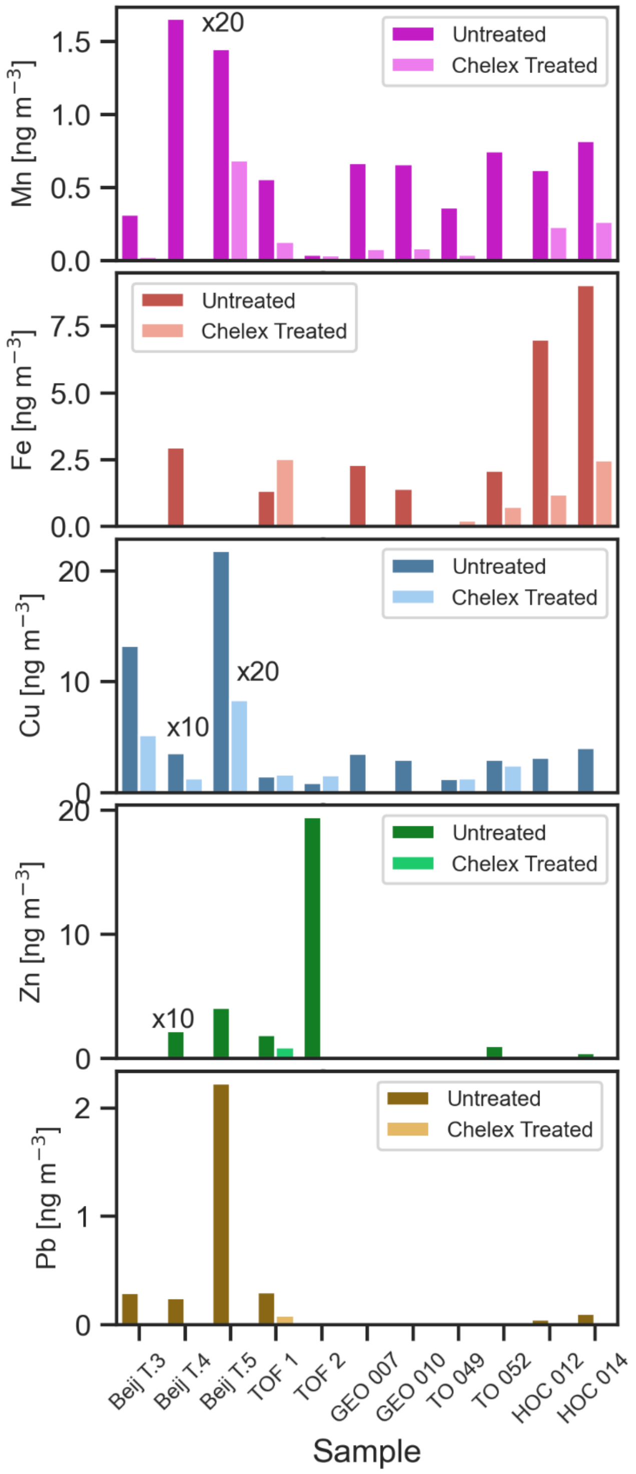

Figure S8. Metal concentration of Mn, Fe, Cu, Zn and Pb for 11 samples before (Untreated) and after Chelex-treatment (Chelex Treated) in ng m^{-3}. For providing a better visibility, the concentrations for Beij T.4$_{Cu}$, Beij T.4$_{Zn}$, Beij T.5$_{Mn}$ and Beij T.5$_{Cu}$ have been divided by the number displayed over the corresponding bars.

XXII, Table S6, Metal-Removal Efficiency by the Chelex Resin

Table S6. Metal-removal efficiency by Chelex resin.

Metal species	mean removal efficiency by Chelex resin	RSD
Mn	84%	19.1%
Fe	91%	15.5%
Cu	91%	15.5%
Zn	85%	24.0%
Pb	85%	24.0%

XXIII, Figure S9, Effect on Organic Compounds by Chelex Resin

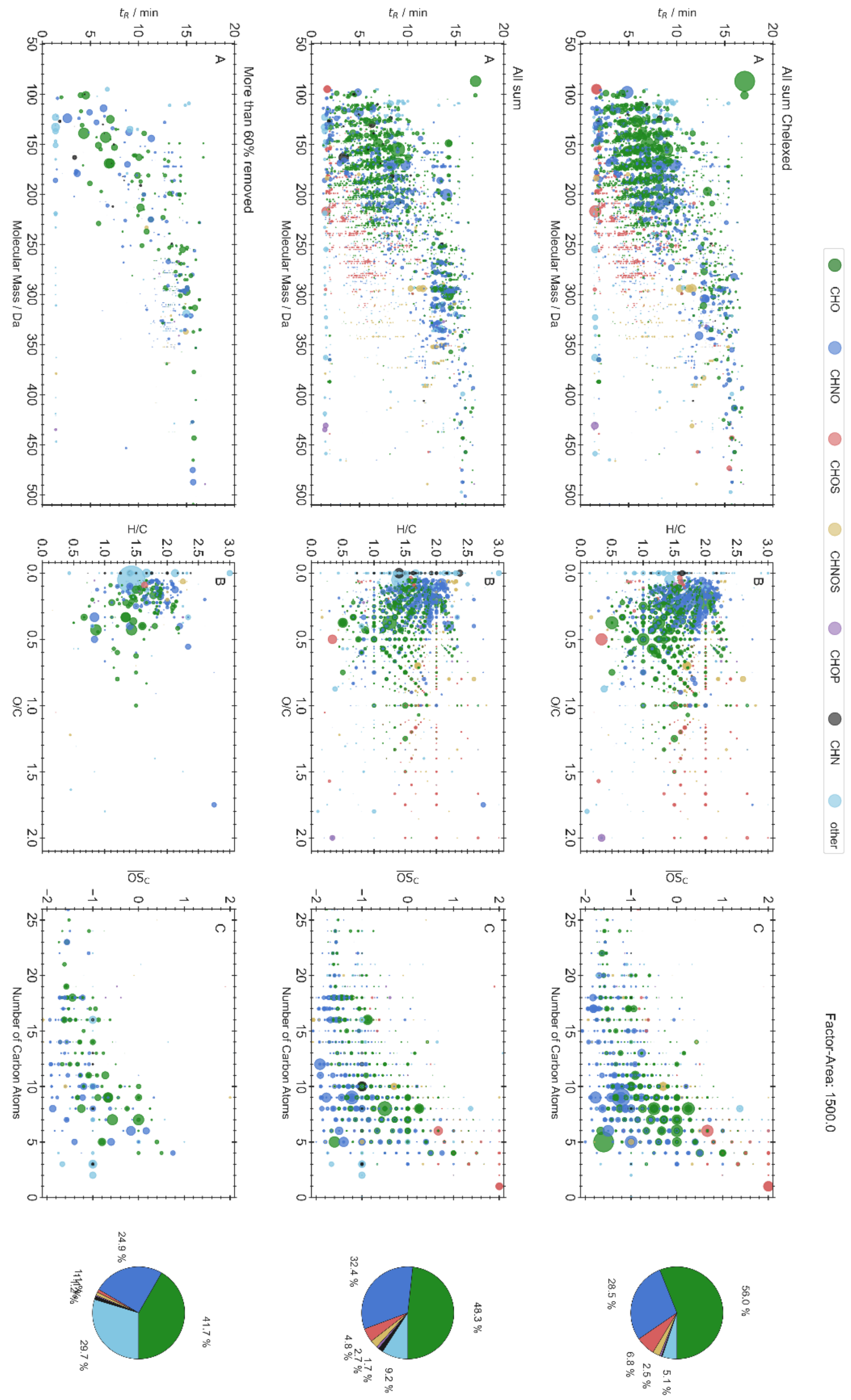

Figure S9. Molecular fingerprint for Chelex-treated and untreated samples and compounds that got removed by more than 60% including A) *m/z* vs. rt, B) Van Krevelen Diagram displaying H/C vs. O/C ratio and C) Kroll plot displaying number of carbon atoms vs. $\overline{OS}_c$ and a pie chart containing the relative contribution of compound groups to the cluster.

XXIV, Figure S10, Backwards Trajectory TO, 29th of April to 7th of May 2022

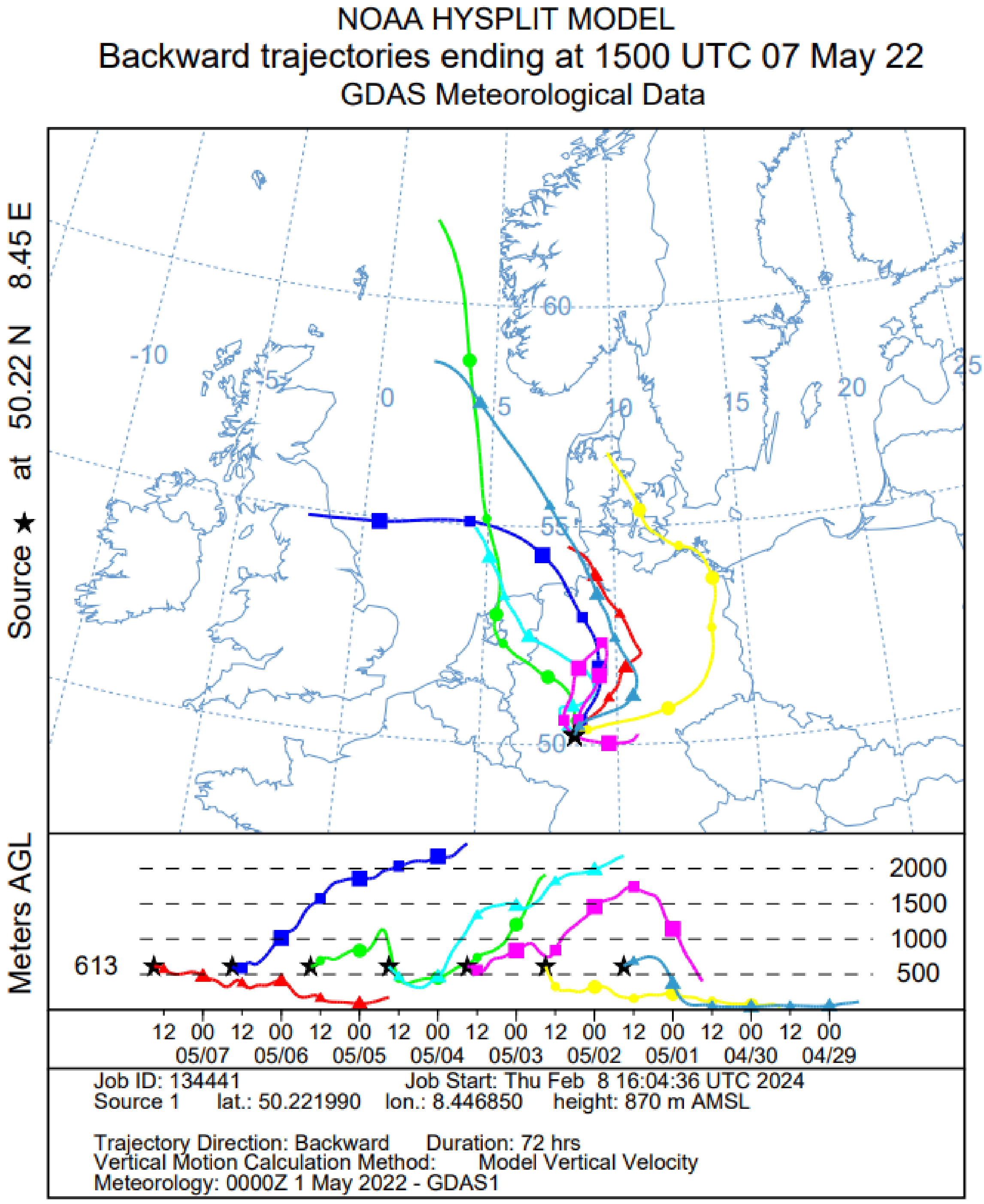

Figure S10. Backwards trajectory, 72 h each, in the period of 29th of April to 7th of May 2022 to Taunus Observatory in the height of 870 m carried out by the NOAA HYSPLIT MODEL.[4]

XXV, Figure S11, Backwards Trajectory TO, 7th to 15th of May 2022

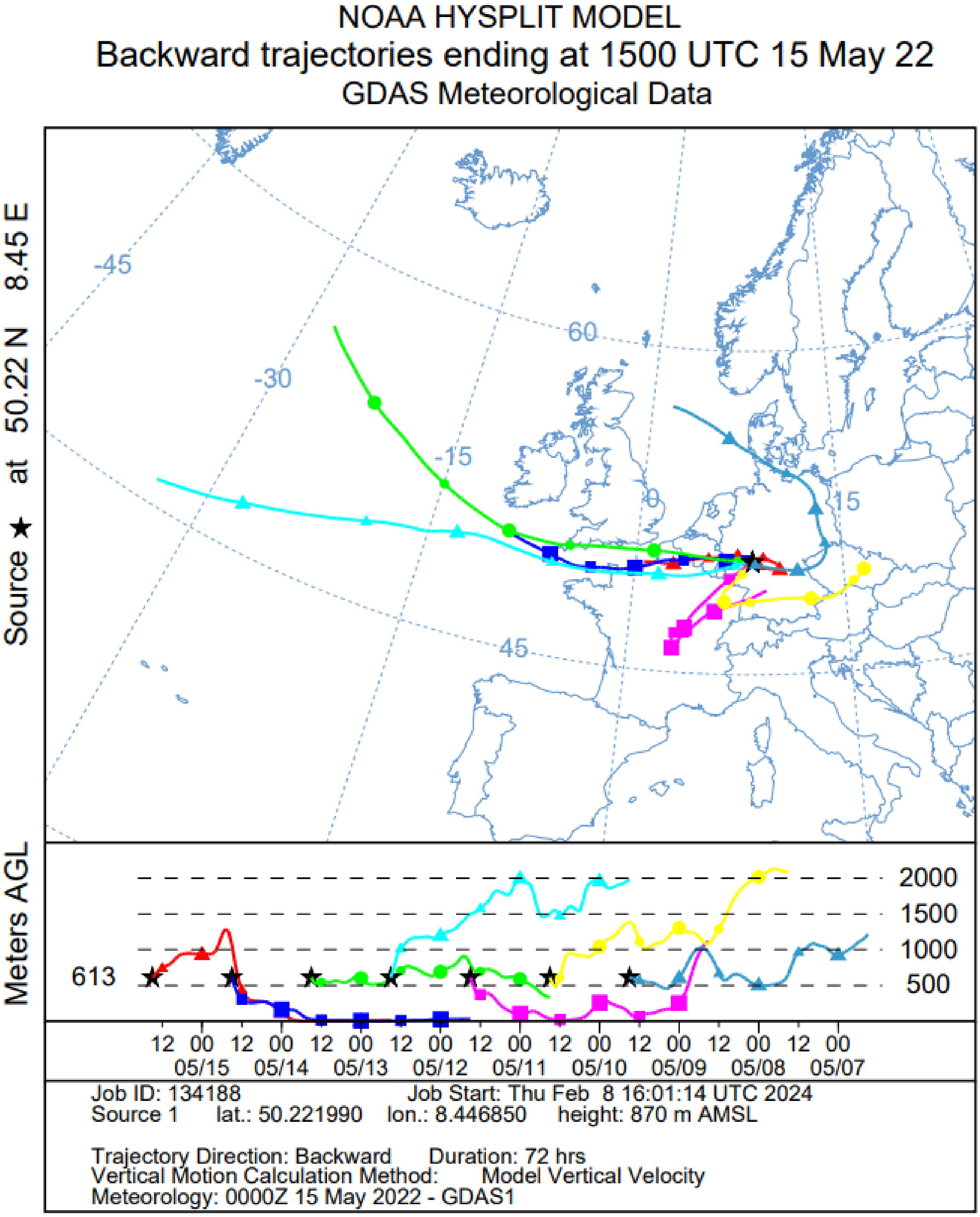

Figure S11. Backwards trajectory, 72 h each, in the period of 7th to 15th of May 2022 to Taunus Observatory in the height of 870 m carried out by the NOAA HYSPLIT MODEL.[4]

XXVI, Figure S12, Measured OP$_M$ for each Location

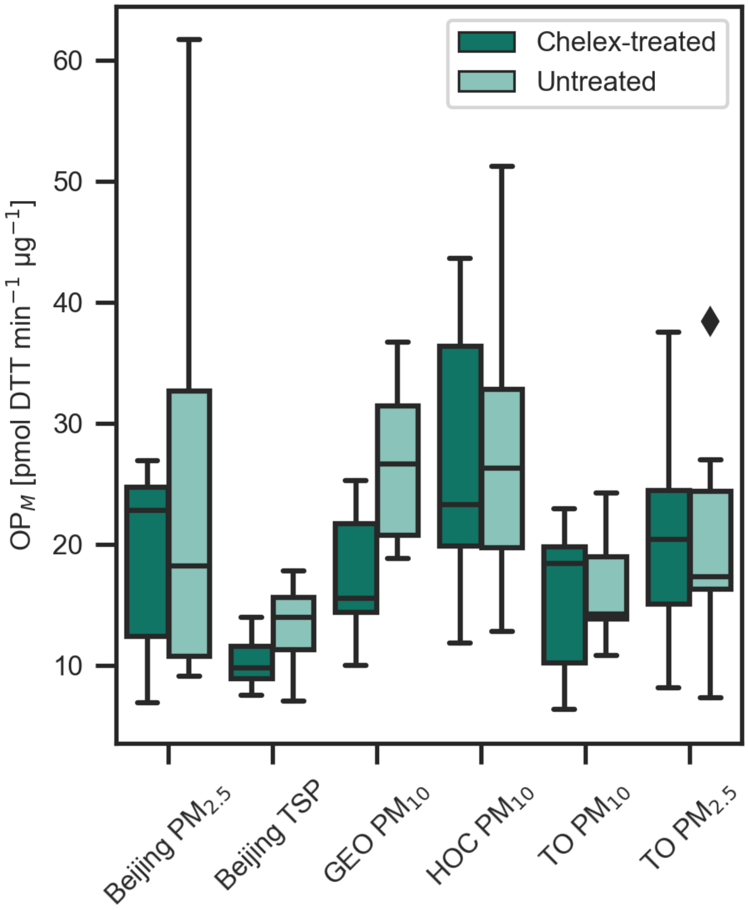

Figure S12. OP$_M$ in pmol DTT min^{-1} µg^{-1} for the locations Beijing, Frankfurt Riedberg (GEO), Frankfurt Hoechst (HOC) and Taunus Observatory (TO), displayed as a Boxplot containing seven samples each. Here, the PM for TO PM$_{2.5}$ and Beijing TSP was estimated based on data from another time, since no data was available. A distinction between Chelex-treated and Untreated is being made. The rhombus mark outliners, which are defined as $x < Q1 - 1.5 \cdot IQR$ or $x > Q3 + 1.5 \cdot IQR$ with $IQR = Q3 - Q1$.

XXVII, Figure S13, Overview of all Measured Parameters for each Sample

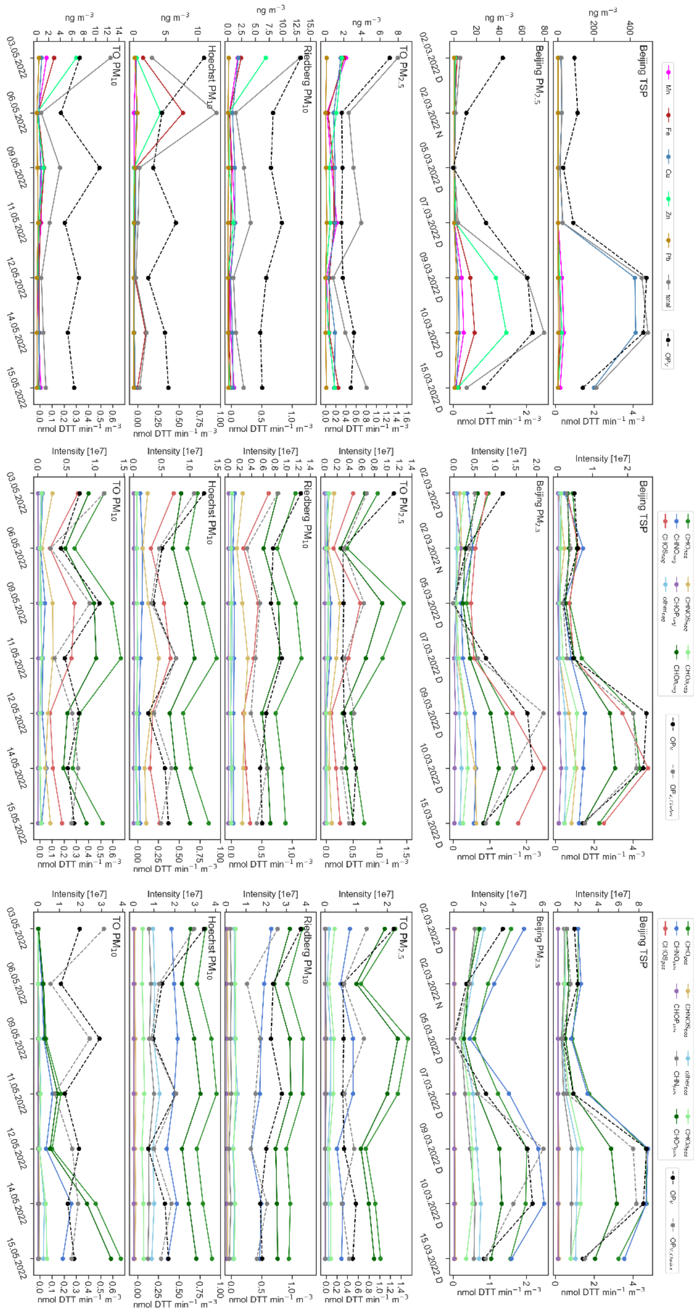

Figure S13. Overview of all samples measured from the locations Beijing TSP, Beijing PM2.5, Frankfurt Hoechst (PM_{10}), Frankfurt Riedberg (PM_{10}), Taunus Observatory PM10 and Taunus Observatory $PM_{2.5}$, by ICP-MS (left), displaying the metal concentration of Mn, Fe, Cu, Zn, Pb and total in ng m-3. In the middle, measurements by UHPLC-HRMS in negative mode displaying the intensity of the compound groups CHO, CHOa, CHOn, CHNO, CHOP, CHNOS,

CHOS, CHOP and other. Right, in positive mode with the additional compound group CHN. In all graphs additionally OP_V and $OP_{V,Cx}$ in nmol DTT $min^{-1}m^{-3}$.

XXVIII, Figure S14, Copper-Removal by Chelex Resin

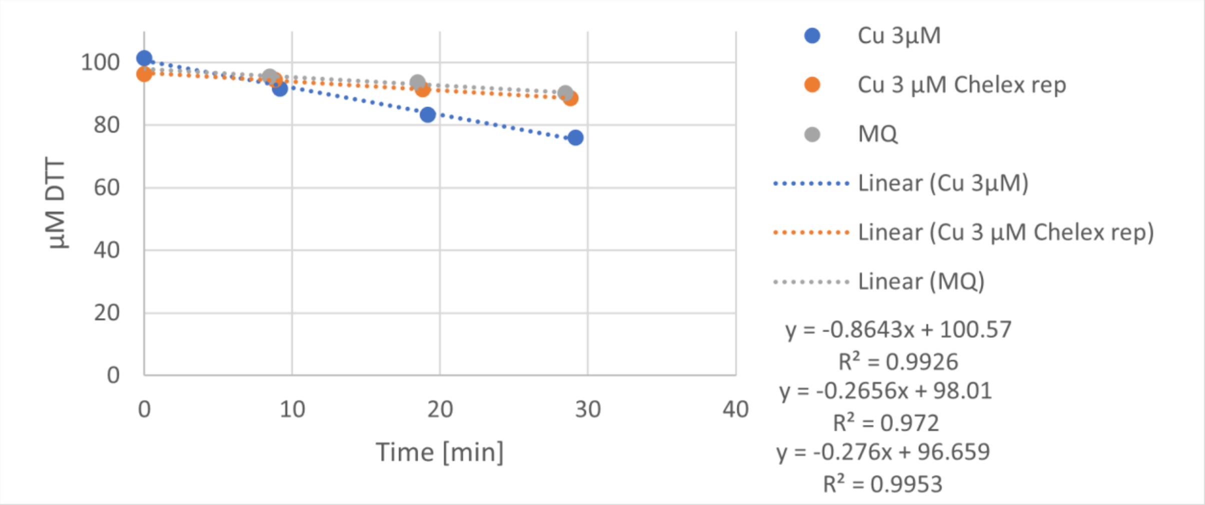

Figure S14. OP caused by copper before and after Chelex-treatment. Cu-consumption rate is comparable to findings by Charrier and Antassio.[5]

XXIX, Figure S15, HCA

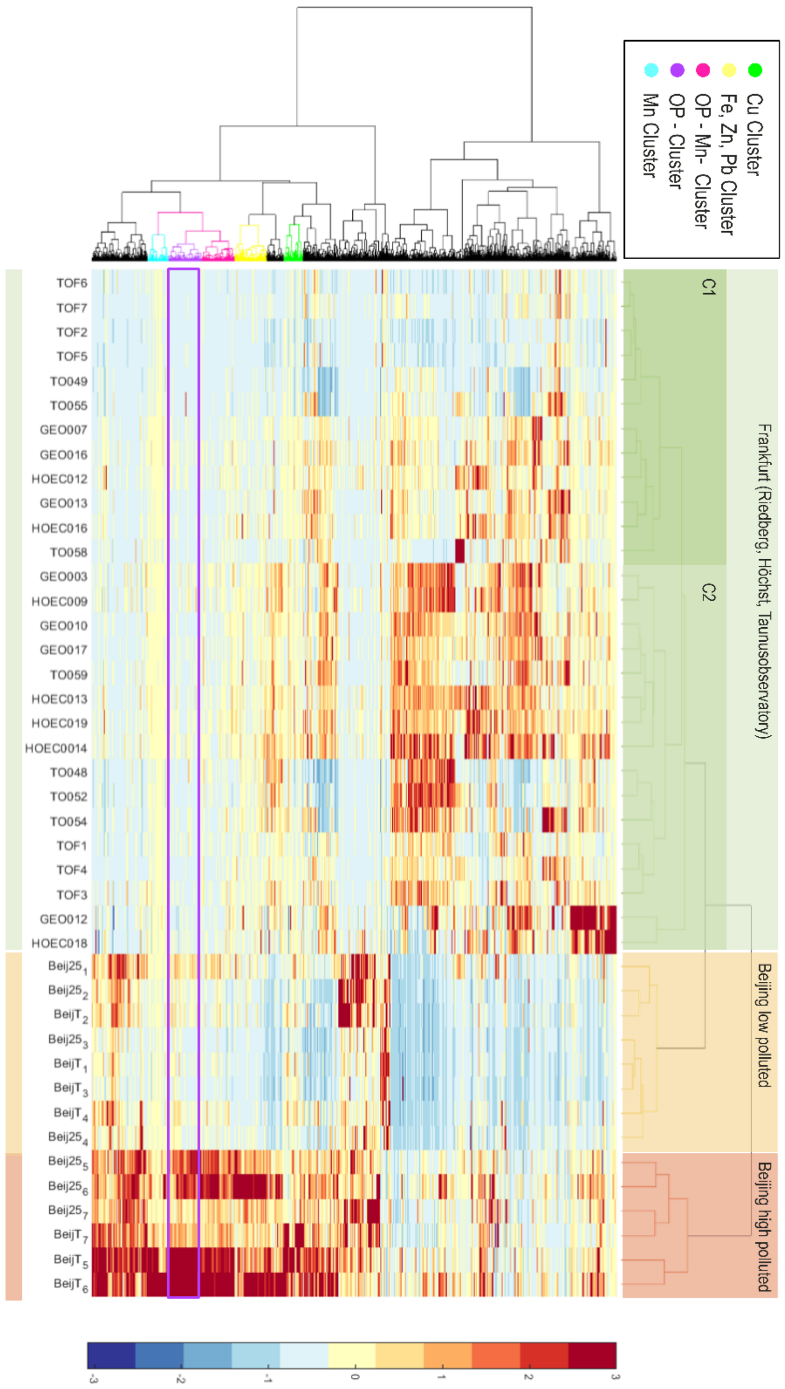

Figure S15. Hierarchical cluster analysis of all samples, metals and OP against all samples measured. Marked are the compound clusters containing copper (green), containing iron, zinc, lead (yellow), containing OP and manganese and OP (pink), containing OP (purple) and cluster containing manganese (blue). The sample clusters are divided into Frankfurt C1, Frankfurt C2, Beijing high polluted and Beijing low polluted.

XXX, Figure S16, MS^2-Spectra of 9,10-Phenanthrenquinone

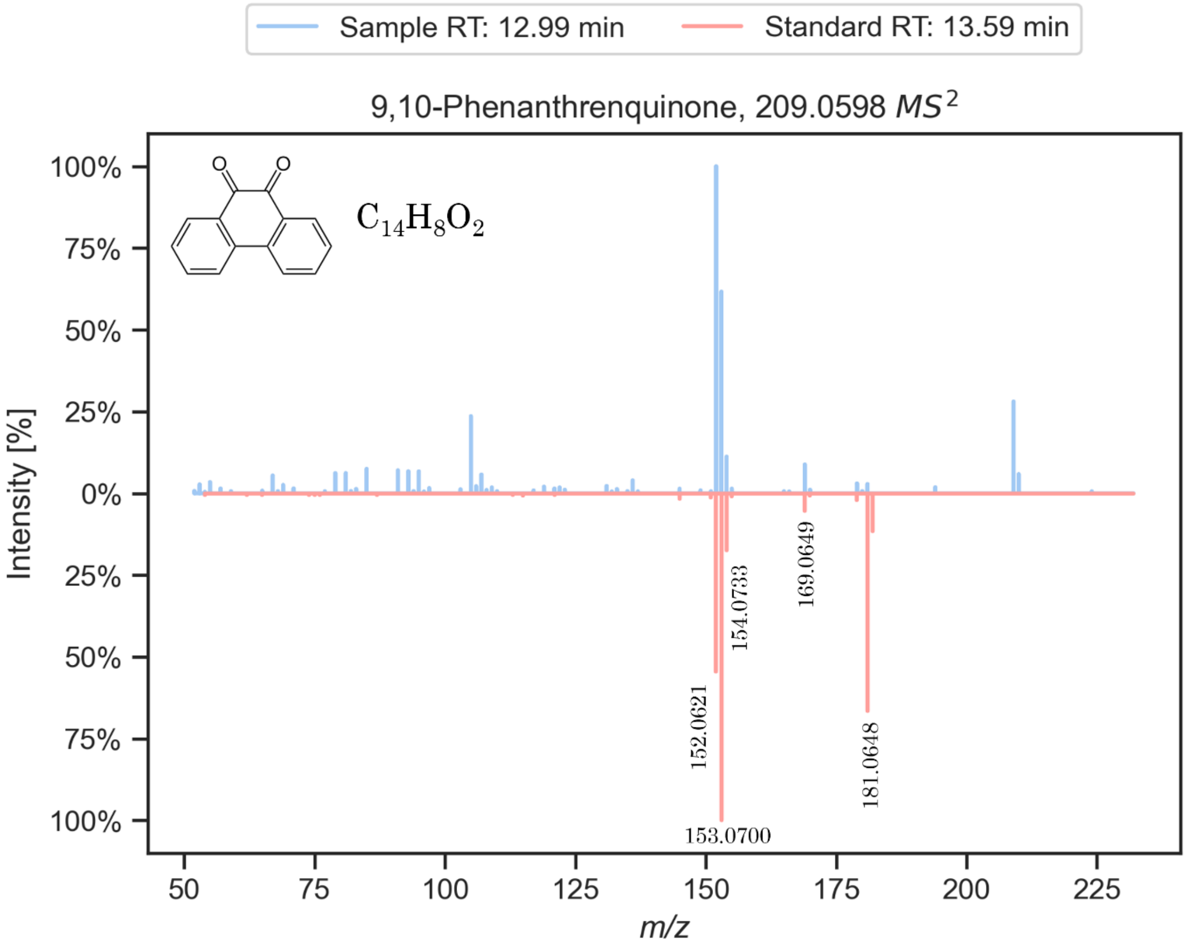

Figure S16. MS^2-Spectra of 9,10-Phenanthrenquinone. Blue: signals found in the sample, red: signals of the standard. Main ions are *m/z*: 152.0621, 153.0700, 154.0733, 169.0649, 181.0648.

XXXI, Figure S17, MS^2-Spectra of Anthraquinone

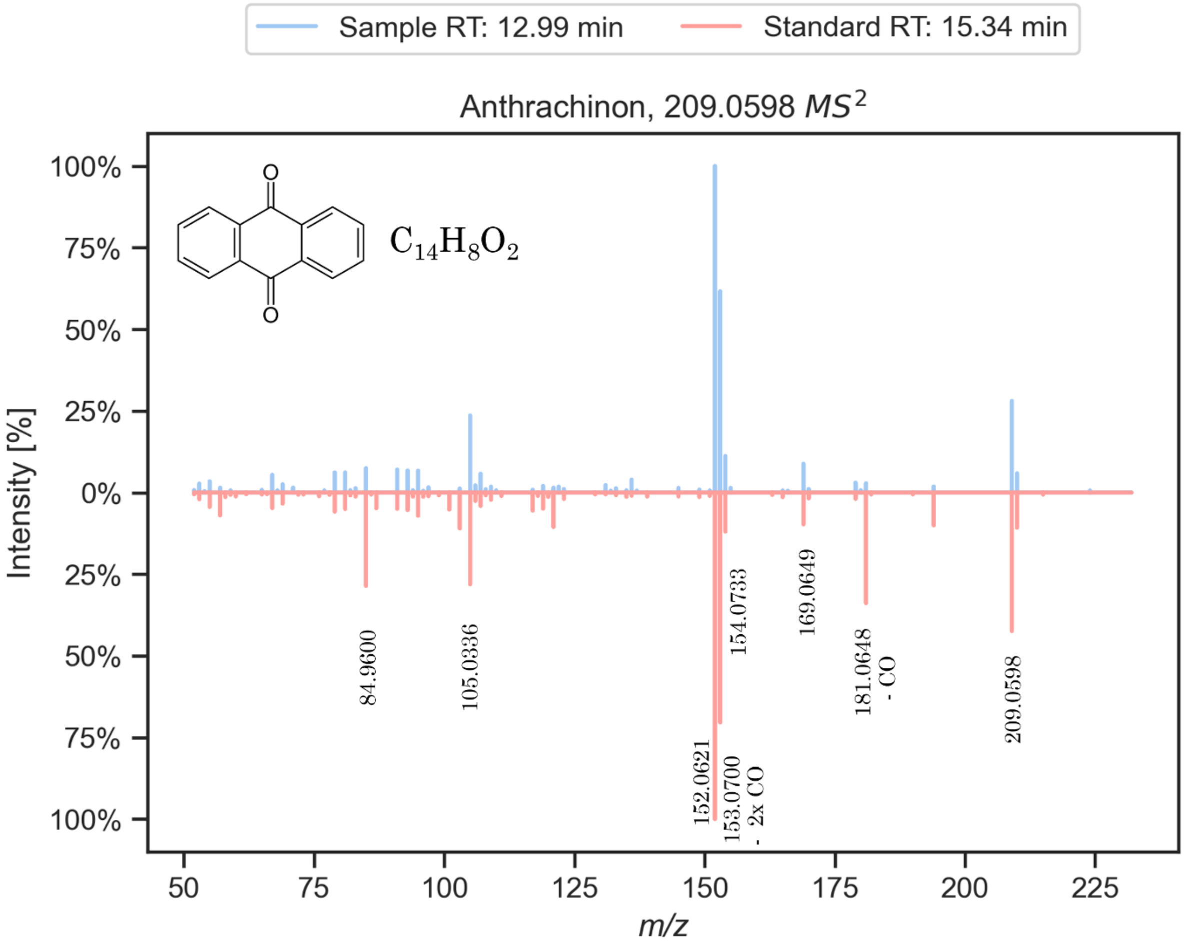

Figure S17. MS^2-Spectra of Anthraquinone. Blue: signals found in the sample, red: signals of the standard. Main ions are *m/z*: 84.9600, 105.0336, 152.0621, 154.0733, 169.0649, 181.0648, 209.0598 (precursor ion).

XXXII, Figure S18, MS^2-Spectra of 1,2-Naphtoquinone

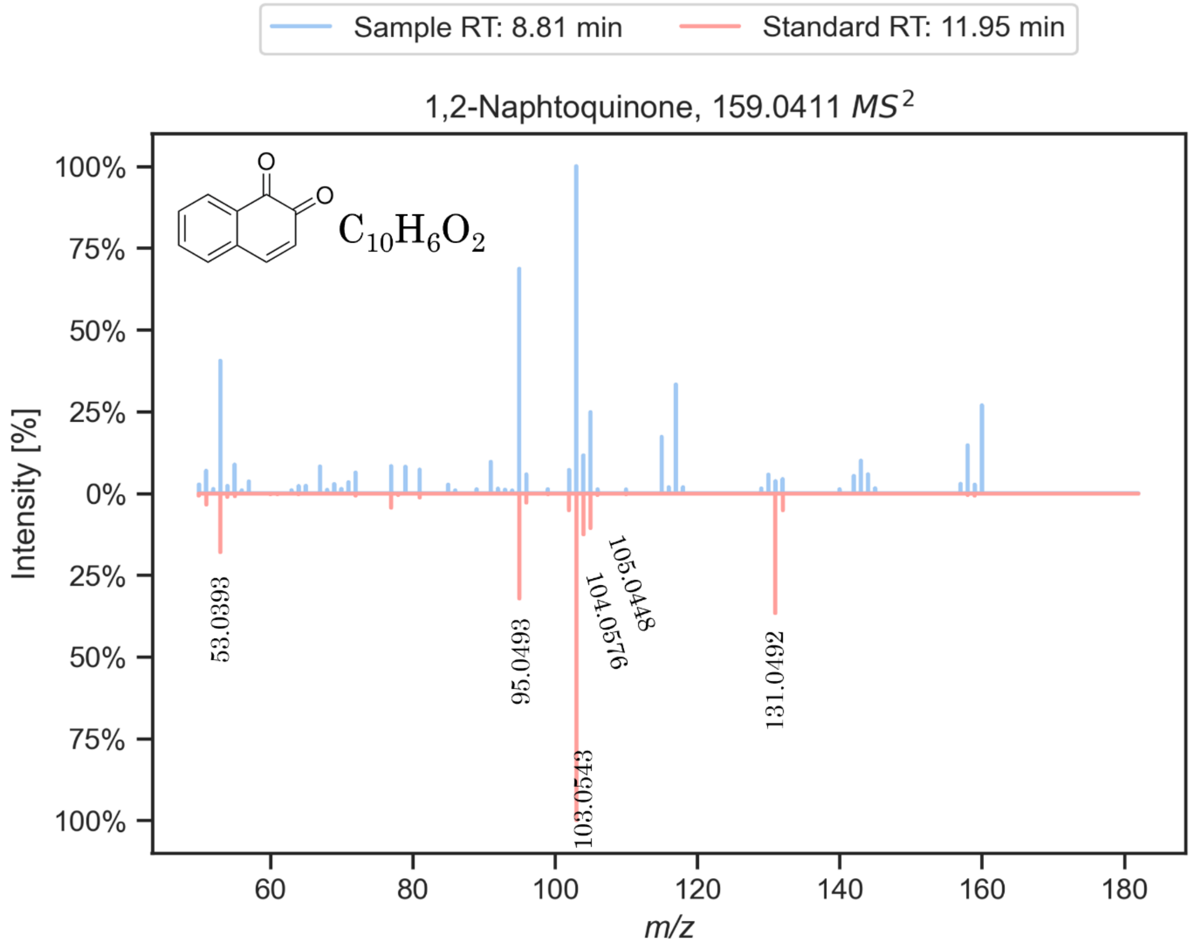

Figure S18. MS^2-Spectra of 1,2-Naphtoquinone. Blue: signals found in the sample, red: signals of the standard. Main ions are *m/z*: 53.0393, 95.0493, 103.0543, 104.0576, 105.0448, 131.0492.

XXXIII, Figure S19, Calibration Curves

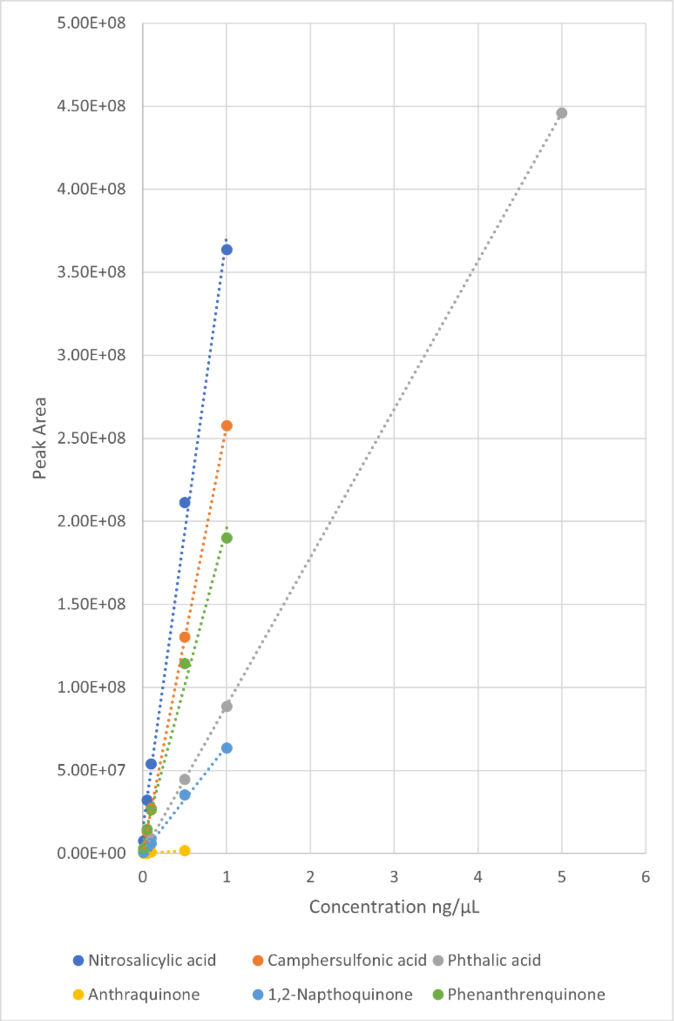

Figure S19. Calibration curves were measured for nitrosalicylic acid (sigma Aldrich, 99%), camphorsulfonic acid (sigma Aldrich, 98%), phthalic acid (Merck, 99.5%), anthraquinone (sigma Aldrich 97%), 1,2-naphtoquinone (Fisher Scientific, 95%), phenanthrenquinone (sigma Aldrich, 99%) using a UHPLC-HESI-HRMS setup.

XXXIV, Figure S20, OP-Activity of Targeted Compounds

A)

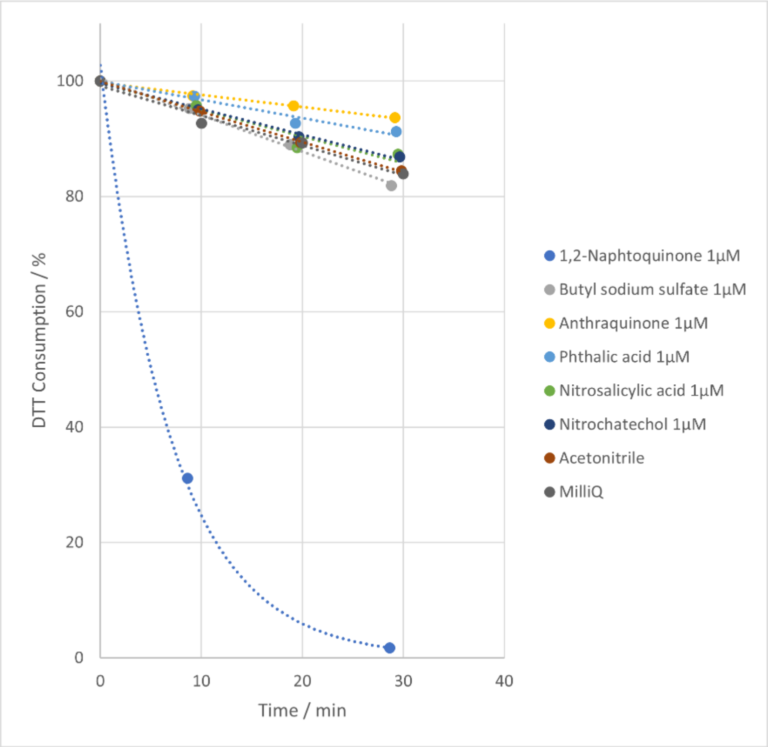

B)

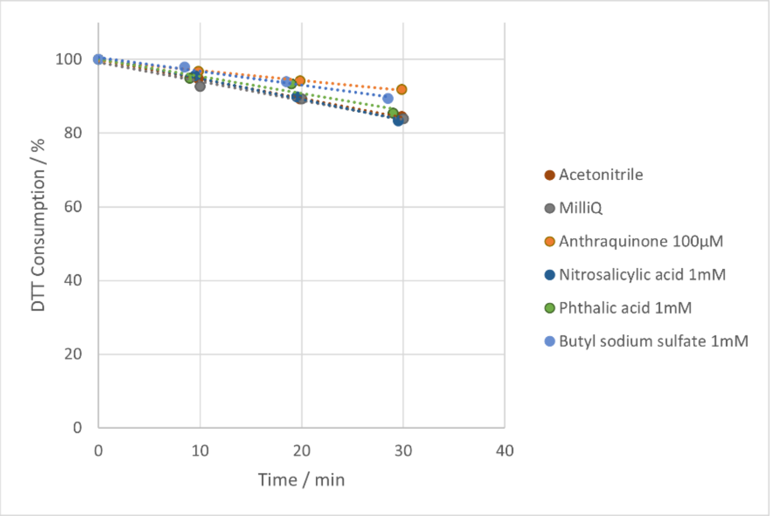

Figure S20. DTT-consumption (%) over time (min) of A) 1µM of 1,2-naphtoquinone, butyl sodium sulfate, anthraquinone, phthalic acid, nitrosalicylic acid and nitrocatechol and B) 1mM of nitrosalicylic acid, phthalic acid, butyl sodium sulfate and 100 µM anthraquinone.

XXXV, Figure S21, Effect of Nitrosalicylic Acid on OP-activity of Quinones

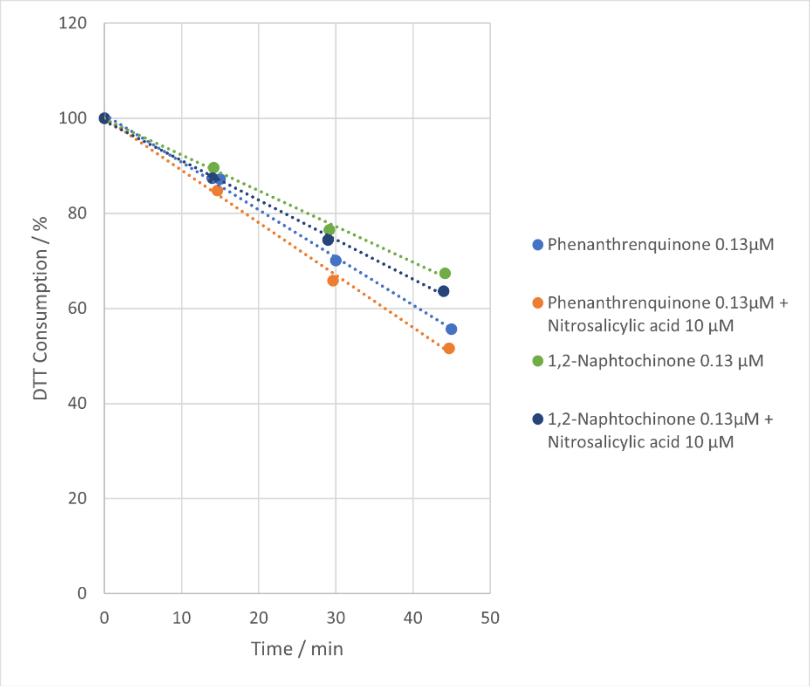

Figure S21. DTT-consumption (%) over time (min) of phenanthrenquinone 0.13 µM, phenanthrenquinone 0.13 µM + nitrosalicylic acid 10 µM, 1,2-naphtoquinone 0.13 µM and 1,2-naphtoquinone + nitrosalicylic acid 10 µM.

XXXVI, Figure S22, Molecular Fingerprints for Sample Clusters

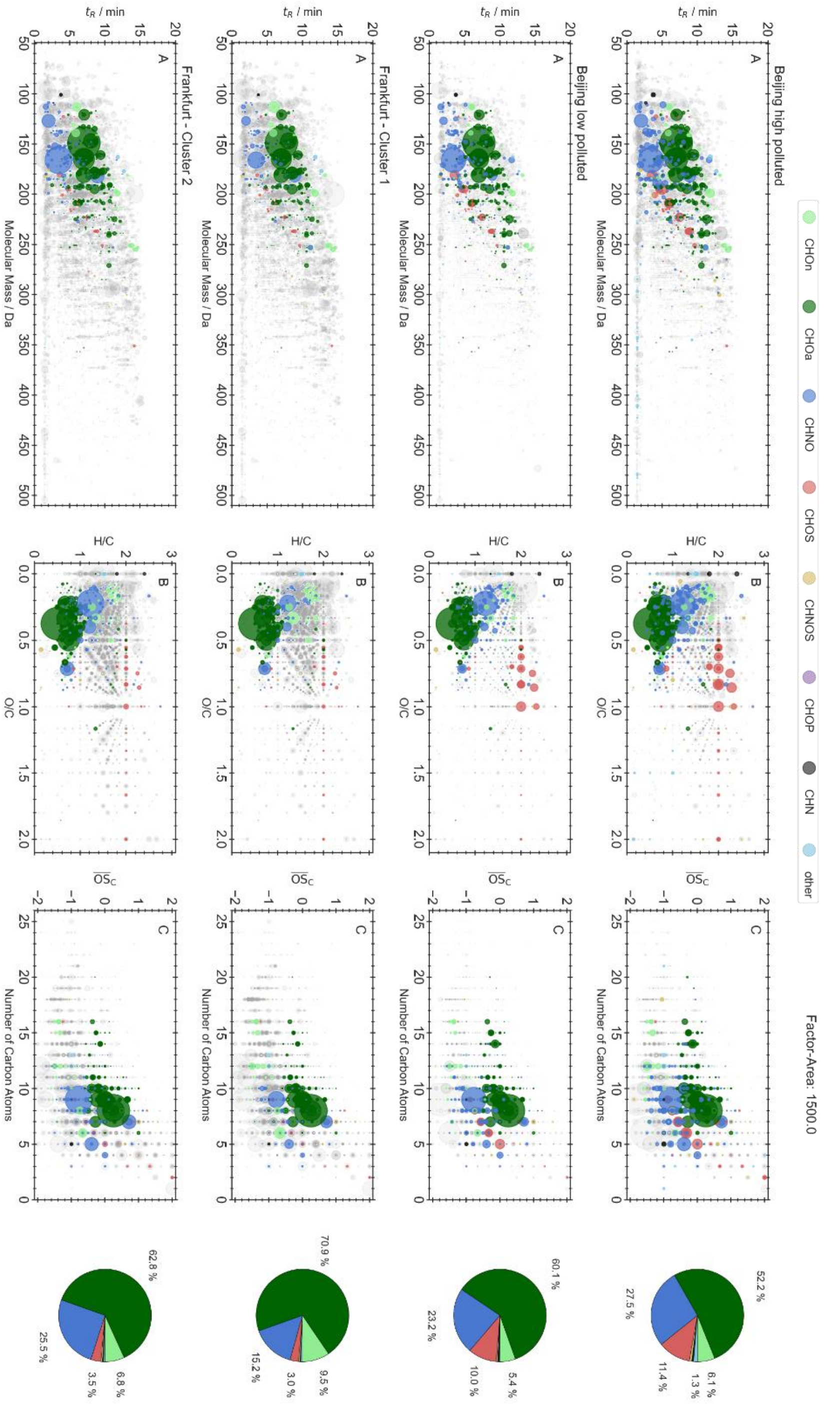

Figure S22. Molecular fingerprint for each sample cluster found in Figure S13 including A) *m/z* vs. rt, B) Van Krevelen Diagram displaying H/C vs. O/C ratio and C) Kroll plot displaying number of carbon atoms vs. $\overline{OS}_c$ and a pie chart containing the relative contribution of compound groups to the cluster.

XXXVII, Figure S23, Backwards Trajectory Beijing, 27th of February to 7th of March 2022

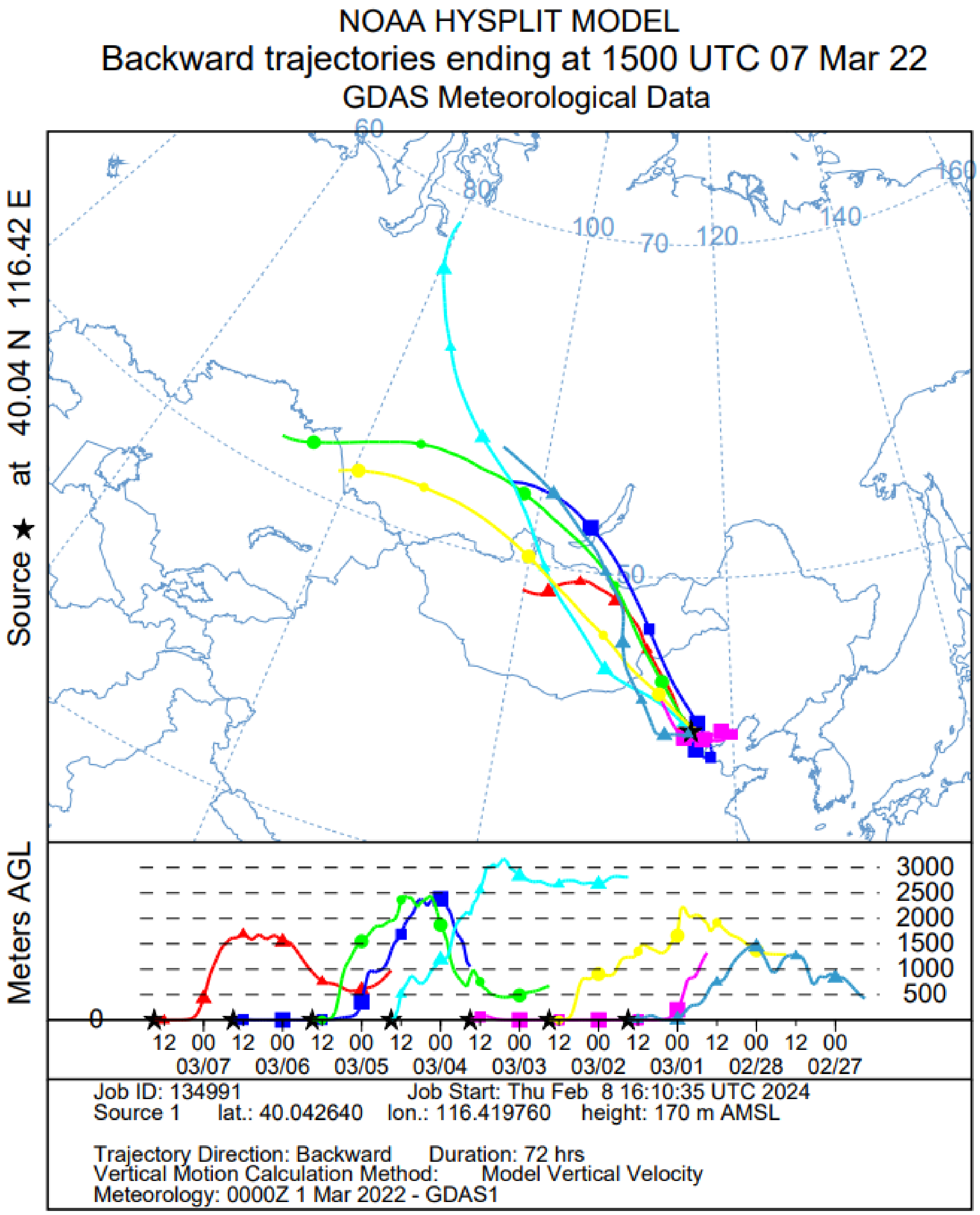

Figure S23. Backwards trajectory, 72 h each, in the period of 27th of February to 7th of March 2022 to Chaoyang, Beijing in the height of 170 m carried out by the NOAA HYSPLIT MODEL.[4]

XXXVIII, Figure S24, Backwards Trajectory Beijing, 7th to 15th of March 2022

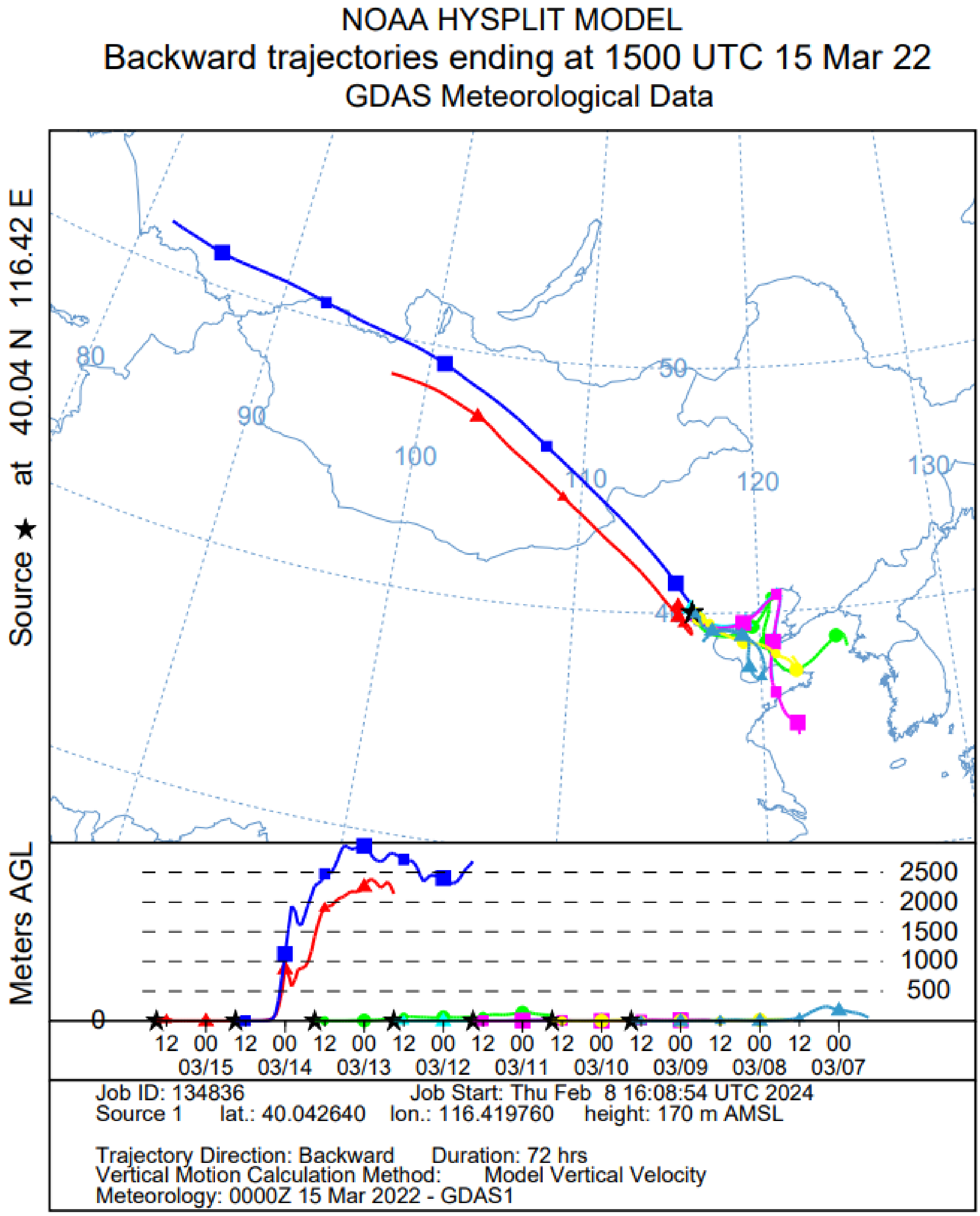

Figure S24. Backwards trajectory, 72 h each, in the period of 7th to 15th of March 2022 to Chaoyang, Beijing in the height of 170 m carried out by the NOAA HYSPLIT MODEL.[4]

References:

(1) Hessisches Landesamt für Naturschutz, U. und G. Luftmessnetz Hessen, 2023. https://www.hlnug.de/themen/luft/luftqualitaet/luftmessnetz.

(2) Government, C. Online Detection Analysis Platform for Airquality, 2023. http://www.aqistudy.cn/.

(3) Yassine, M. M.; Harir, M.; Dabek-Zlotorzynska, E.; Schmitt-Kopplin, P. Structural Characterization of Organic Aerosol Using Fourier Transform Ion Cyclotron Resonance Mass Spectrometry: Aromaticity Equivalent Approach. *Rapid Commun. Mass Spectrom.* **2014**, *28* (22), 2445–2454. https://doi.org/10.1002/rcm.7038.

(4) Stein, A. F.; Draxler, R. R.; Rolph, G. D.; Stunder, B. J. B.; Cohen, M. D.; Ngan, F. NOAA's HYSPLIT Atmospheric Transport and Dispersion Modeling System. *Bull. Am. Meteorol. Soc.* **2015**, *96* (12), 2059–2077. https://doi.org/10.1175/BAMS-D-14-00110.1.

(5) Charrier, J. G.; Anastasio, C. On Dithiothreitol (DTT) as a Measure of Oxidative Potential for Ambient Particles: Evidence for the Importance of Soluble Transition Metals. *Atmospheric Chem. Phys.* **2012**, *12*, 11317. https://doi.org/10.5194/acpd-12-11317-2012.
